# Collective action problems led to the cultural transformation of Sāmoa 800 years ago

**DOI:** 10.1371/journal.pone.0304850

**Published:** 2024-06-20

**Authors:** Ethan E. Cochrane, Seth Quintus, Matthew Prebble, Ta‘iao Aumua Ausilafa‘i Matiu Tautunu, Dolly Autufuga, Mana Laumea, Alexandra Queenin, Paul Augustinus, Noa Kekuewa Lincoln

**Affiliations:** 1 Anthropology, School of Social Science, University of Auckland, Auckland, New Zealand; 2 Department of Anthropology, University of Hawai’i at Mānoa, Honolulu, Hawaii, United States of America; 3 School of Earth and Environment, University of Canterbury, Christchurch, New Zealand; 4 Department of Archaeology and Natural History, School of Culture, History and Language, The Australian National University, Canberra, Australia; 5 Centre for Samoan Studies, The National University of Sāmoa, Le Papaigalagala Campus, To‘omatagi, Apia, Sāmoa; 6 Tropical Plant and Soil Sciences Department, College of Tropical Agriculture and Human Resources, University of Hawaiʻi at Mānoa, Honolulu, Hawaii, United States of America; 7 School of Environment, University of Auckland, Auckland, New Zealand; Utah State University, UNITED STATES

## Abstract

In this research we identify the processes leading to hierarchical society in a region of Sāmoa, the often-labelled ʻbirthplace’ of the Polynesian chiefdoms. Our analyses in the Falefa Valley on ʻUpolu island combine lidar mapping and ground survey to reveal an extensive system of archaeological features: rock walls, ditches, and platforms. Excavation and radiocarbon dating underpin a feature chronology and characterize feature variation. Soil nutrient analyses and geoarchaeological coring indicate spatial differences in the agricultural potentional of the valley and human modification of the environment over time. Our results demonstrate that the construction of large rock walls, some several hundred meters long, began approximately 900–600 years ago, shortly after rapid population rise in Sāmoa. This was followed by the building of small rock walls, often enclosing rectilinear fields or platforms. Both rock wall types are concentrated in the western and northern regions of the valley and greater rock wall densities are associated with areas of higher agricultural potential. The earliest wall construction was penecontemporaneous with partial forest removal that created a more productive wetland environment in the southeastern region of the valley, an area later a focus of agricultural ditching. We propose that with population rise the variable fertility of agricultural land became a significant resource gradient, influencing the population in two ways. First, areas of more fertile agricultural land promoted territorial behaviour, including large rock walls, and led to a collective action problem. Second, niche construction in the form of human-induced environmental change created a productive wetland agricultural system that was enhanced with a reticulate ditch network, the maintenance of which also led to a collective action problem. We conclude that in the Falefa Valley, the second largest catchment in Sāmoa, collective action problems were the cause of increased social hierarchy and may underlie the origins of chiefdoms throughout Polynesia.

## Introduction

For archaeologists and anthropologists two cultural innovations have long been considered catalysts for the development of complex chiefdoms in Polynesia: intensified agriculture and changes in land tenure, including the development of territoriality [1–4]. These innovations, often tied to the elite mobilization of resources, wealth or surplus production, are also correlated with the rise of hierarchical society across much of the world [3, 5], despite much variation in how societies are structured [6]. Ethnohistorically, in Polynesia and other parts of the Pacific, control of both land and the surplus produced by intensified agriculture was often passed down through chiefly lines. Although the specifics vary with time and place, the transference of this chiefly control from person to person could be negotiated by members of the lineage [e.g., in Sāmoa, 7] or by stricter genealogical rules [e.g., in Hawaiʻi, 8]. Such transmission of control and wealth within a subset of a larger population is a foundational characteristic of hierarchical society [9].

Throughout the Pacific, where good data are available, increasing social hierarchy is also associated with population increase [e.g., see chapters in 10], and this increase may be tied to greater territoriality and resource competition [11, 12], which can drive both conflict and attempts to enhance agricultural surplus [8, 13]. Territoriality may not only be in the form of potentially violent conflict, though that is well documented in the region [14], but also includes the bounding and physical demarcation of land which highlights emergent changes in how land and land tenure is conceptualized [15, 16].

**Fig 1:**
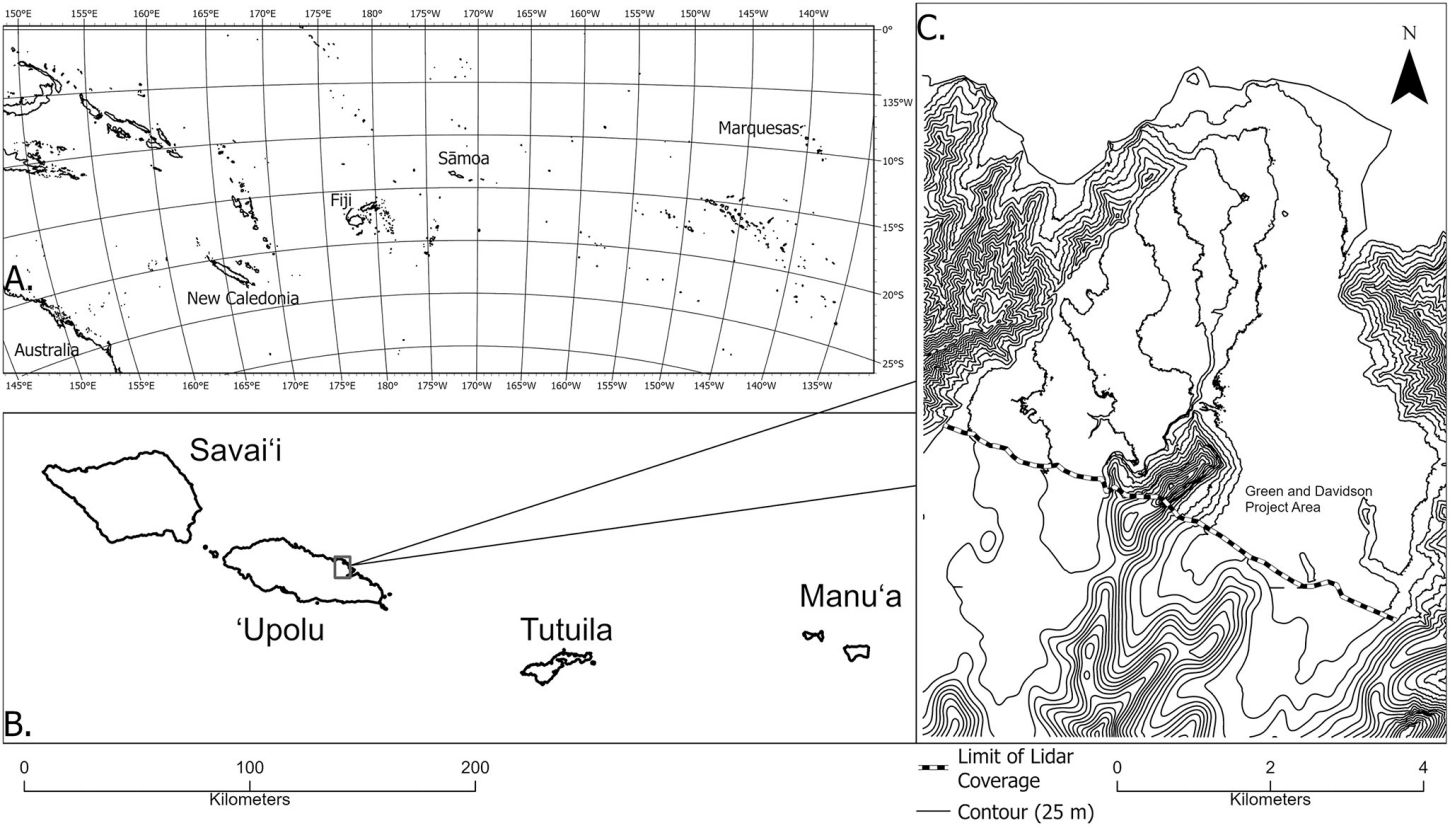
Maps of the Pacific Islands (A), Samoa (B), and the Falefa Valley (C).

These concepts of land tenure and agricultural intensification are relevant to archaeological explanations of the cultural transformation of Sāmoa over the last millennium. Our work in the Falefa Valley on the island of ʻUpolu (Fig 1) has identified the construction of large, linear rock structures beginning approximately 800 cal BP. A dramatic rise in population, probably from in-migration [17], occured just before this. Within a few hundred years of these changes, construction of small rock walls begins, as well as forest clearing and ditching in a newly created wetland environment. We propose these interconnected changes are explained by selection for both territorial [12] and niche construction behaviors [18] that increase agricultural production. These behaviors also engendered collective action problems, situations where an indvidually-costly behaviour may benefit a group [19], and increasing social hierarchy. We argue that population increase was the trigger for these changes.

### The development of chiefdoms in Polynesia: Land, territories, and inheritance

Land and agricultural resources are fundamentally tied to leadership in Polynesia and much of the Pacific [2, 20, 21]. In a practical sense, both land and agriculture provide opportunities for leaders to mobilize resources to support a range of activities–community works, public rites including feasting, gifting, and others–that are the responsibility and expectation of leadership positions. This links chiefs to key components of ideology in the region, specifically *mana* [22], which is thought to have a deep history in Polynesia [23]. *Mana*, a form of efficacy that is genealogically inherited and ethically mediated [24, 25], is both a marker and key trait of leadership. Food and general abundance symbolize the presence of *mana* [21, 22, 26].

Exploring variation in both agricultural behaviors and the lands whereupon they are practiced is therefore key to archaeological explanations of ancient social change. We use evolutionary and ecological theory to generate expectations of human behavior and its empirical results within the context of heterogenous agricultural lands and practices, growing populations, and intergenerational cultural inheritance [9]. Both economic defensibility and ideal despotic distribution models posit that territoriality is advantageous when resources, including fertile agricultural lands, are predictable, dense, and unevenly distributed [12, 27, 28]. In these situations, investments in territoriality can be expected as exclusive access can enhance both resource availability and social power. The latter occurs as those individuals in less favorable environmental positions require the aid of those in more favorable positions, giving rise to relationships of inequality. In Polynesia, this process would seem to reinforce the ideology of *mana* as leaders who are able to define and hold exclusive access to productive lands are more likely to fulfill the expectations of their position [16]. As Valeri [26] further notes, the belief in one’s *mana* results in a form of positive despotism [after 29] wherein labor is attracted to power, which, again, makes it more likely that a leader can provide what is expected.

The archaeological presence of territorial behavior, then, is an important signifier of social conditions. It marks a change in land tenure, highlighting an increase in exclusive access and control. While knowledge of land claims with physical boundaries was no doubt present in the deeper past, the tangible nature of physical boundaries affects their inheritance, a process that builds upon emergent inequality through time [9]. Physical boundaries are retained on the landscape through ecological inheritance [30], promoting the continuation of exclusive access. As land is generally held by descent groups in Sāmoa, and most other places in Polynesia [20], the intergenerational inheritance of physically bounded and spatially heterogeneous land can act to enhance social difference through time. In this way, the temporal patterns and spatial context of territorial behavior, even simple boundary walls, provide important insights into the growth of chiefdoms in the region. Unfortunately, such data are missing or understudied for swaths of Polynesia, including the large islands of Sāmoa.

### Sāmoa in context

Sāmoa was first settled between 2900 and 2700 cal BP [31, 32] by populations leaving the circum-New Guinea islands. These populations arrived in the southwestern Pacific, the islands from Vanuatu in the west, stretching to Sāmoa and Tonga in the east. Domestic activities mark the earliest use of land in Sāmoa, with small, and perhaps isolated [33], coastal occupations appearing soon after settlement, and in island interiors several hundred years later [34, 35]. Larger settlements on the coast and inland appear after about 1000 cal BP [36, 37], but some probably began earlier as smaller settlements [38]. New social relations around 1000 cal BP are also indicated by the construction of ceremonial platforms and mounds [37, 39–41]. And while researchers have debated such settlement variation in terms of population size change [33, 42–44], a recent genetic analysis has now provided new insights.

Harris et al. [17] analyzed the whole genomes of almost 1200 contemporary Sāmoans using identity-by-descent measures, a technique that compares the length of genetic segments that are similar due to descent. As both population size and generational time influence the length of descent-similar segments, segment length distributions can be used to estimate these parameters. Harris et al. concluded that Sāmoa was first settled by an extremely small population [predicted by 33, 45] that experienced consistent bottlenecks [predicted by 46] until about 1000 years ago (30–35 generations) at which point there was a rapid rise in population.

Variation in land tenure and use that correspond to changing settlement patterns is modestly documented with several case studies of landscape-scale stone and earth architecture [47–50]. For Jennings et al. [50], these landscapes are the manifestation of what they called household units (HHU) that define the land of extended family groups. Jennings et al. grouped these HHU into wards, typified by the site of Mt. Olo in western ‘Upolu [51]. In the eastern Manuʻa islands of the archipelago (see Fig 1), semi-nucleated communities of habitation and agricultural terraces have been documented along with associated agricultural infrastructure (e.g., ditches, walls) within the confines of those communities [36]. It has not yet been possible to distinguish archaeological categories comparable to HHU or wards in these locations, though the presence of physical boundaries in the form of ditches and linear mounds indicates a concern with land access [52]. Ditching across the western islands has only recently been mapped in detail [but see 53], likely related to flood control and land divisions [54–56]. The presence of these features suggests further concerns with agricultural intensification and land control.

Reliable and detailed chronologies of the built landscape are limited in Sāmoa [52]. Boundaries that probably demarcate lands for different uses are confined to the last millennium [57]. In particular, rock-walled and ditched areas as well as artificial terraces do not appear in number until about 1000 cal BP [58]. The clearest picture of past changes to land-tenure emerges from recent work on the Manuʻa islands. On the island of Taʻu, formal land boundaries are relatively late in the precolonial sequence, appearing around 300 cal BP [52], less than 100 years before Samoans first encoutered European sailors. On Ofu, land boundaries appear around 1000 cal BP, but these are few and associated with individual habitation terraces. Later infrastructure, in the form of networks of ditching, that reflects suprahousehold authority stems from 550 cal BP and later.

Little is also clear about Sāmoan agricultural change over the last three millennia [58]. While the first Sāmoans removed and modified forests to cultivate crop plants [59–61], there is no clear evidence of specific taxa grown. It seems likely, however, that the main four cultigens recorded historically [62]–taro (*Colocasia esculenta*), breadfruit (*Artocarpus altilis*), banana (*Musa* spp.), and coconut (*Cocos nucifera*)–were present from early on. In addition to shifting cultivation, Sāmoans built agricultural infrastructure as described above; terraces, ditches, and rock walls were all used [58]. Past agricultural practices also changed through expansion and intensification on all major islands of the archipelago, but scant attention has been paid to the timing and causes of these changes.

### Environment of the Falefa Valley

The Falefa Valley (see Fig 1) is on the north coast and eastern half of the island of ‘Upolu. The valley is roughly 10–11 km at its longest extent and about 5.5 km wide, with two branches diverging inland, west and east, separated by an escarpment. The valley is formed by the Fagaloa volcanic series ranging in age from 2.78–1.37 Ma [63], but younger sediments of the Mulifanua volcanic series overlie this throughout the western arm of the valley and while the precise ages for these volcanics are not well documented, they are likely to be < 1 Ma [63]. In much of the eastern arm of the valley the Fagaloa volcanics are overlain by the Holocene-aged Lalomauga Alluvium [64]. Finally, the southeastern portion of the valley floor is an area of lower elevation and we refer to it as the Falevao basin, after the village located there.

Little research has been conducted on the agricultural potential of the Falefa Valley. ‘Upolu-wide analyses by Schroth [65], for example, suggest that soil fertility decreases as one moves inland from the coast, but these analyses included only one sample location in the Falefa Valley [see 39]. More recent work by Terry et al. [66] documented an extremely high sedimentation rate in the Falevao basin, approximately 4 cm/year over the last 40 years, and they attribute this to storm-derived alluvial flooding.

Perennial rivers flow through both sides of the valley, and a stream crosses the middle of the valley connecting both watercourses. These rivers and the higher elevation landscape in the western half of the valley (see Fig 1) have created waterlogged sediments and high rates of sediment deposition in the Falevao basin. In contrast, most of the western half of the valley has shallower free-draining sediments (excepting a section of Lalomauga Alluvium in the southeast).

Precipitation rates in the valley derived from limited rain gauge data (http://www.samet.gov.ws/) and satellite imagery [67] indicate high interannual variability, but with an annual average rainfall between 2.5 m towards the coast to 6 m at higher elevation. Rainfall on ‘Upolu is derived primarily from orographic precipitation, formed along the west-east montane ridgeline that rises to up to ~1110 masl, combined with the seasonal extension of the South Pacific Convergence Zone [68]. Compared with other islands in West and East Polynesia, the inter- and intra-island seasonality and interannual hydroclimate of Sāmoa is relatively muted. Under extreme El Niño years in the Falefa Valley, hydroclimate conditions might limit dryland agriculture, but the sediments in the Falevao basin remain water-logged from perennial river flow and hillslope seepages. During high precipitation years, or even under average annual conditions, the Falevao basin is highly flood prone. Taro (*Colocasia esculenta*) is cultivated in these water-logged sediments today, even though the ancient ditches are not typically maintained.

### Archaeological features in an agricultural context

Green and Davidson [69] selected the Falefa valley for archaeological field work because it was thought to be a relatively optimal place for human settlement throughout the cultural sequence [70:161]. However, Davidson [71] reconsidered this view more recently given the vulnerability of large sections of the valley to flooding. The original archaeological investigation of Falefa focused on the southeastern quadrant of the valley, principally along the talus slopes, though additional areas on the valley floor were also investigated [69:2]. The seaward extent of the investigation was nearly 2 km inland from the coast and the survey extended to the upper boundary of the eastern section of the valley. The types of archaeological features identified by Davidson and their distribution were variable across the project area, reflecting at least some underlying environmental variation. A range of feature types was recorded, but we highlight three of these features here relevant to our research: ditches, walls, and mounds/platforms. While never explicitly defined by Davidson [53], Table 1 lists descriptions of these features and their inferred use.

**Table 1:** Archaeological feature descriptions and uses summarized from [53].

Feature	Definition	Previously Proposed Uses
**Wall**	artificially raised linear structure made of stone, earth, or a combination of the two	Land boundary, walkways, agronomic features
**Ditch**	artificially constructed linear depression situated below average level of the ground surface	drainage for agricultural activities, walkways
**Mound/Platform**	artificially raised rectangular, rounded, or squared structure with free standing sides around entire perimeter. Top surface can be slightly convex (mound) or flat (platform)	Foundations for superstructures (e.g., houses, community houses, temples) or base for activities (e.g., pigeon catching)

Ditches were documented on the eastern slopes of the valley and were noted on the valley bottom, though the latter were not formally recorded [70, 72]. Ditches were inferred to have an agricultural function, serving as drainage devices that demarcated raised beds [see, e.g., 73].

Rock walls were also found in a few locations, from the ridges to the valley bottom [70:156, 74:7–10]. These were described as meandering features that defined spaces around platforms and mounds, served as the boundaries of household or larger social units, delimited paths, sometimes with a level or sunken area between parallel walls [53:238], and enclosed agricultural plots [70:157]. Significant variation in construction existed within this feature class, including distinctions between large causeways sometimes with wide flat top surfaces, parallel double walls bounding a pathway, and smaller stacked-rock walls.

Mounds or platforms were more rarely observed, with the highest number documented along the northwestern extent of the original project area [75]. The relative lack of mounds or platforms observed in the Falefa Valley is somewhat unique, for in other valleys these features are abundant [see 70:161–162]. In fact, the general abundance of mounds has now been well documented across ‘Upolu and on Savai‘i [37, 39, 49–51].

## Methods

To answer questions about the roles of land tenure change, territoriality, and agricultural innovation, we initiated a landscape study using lidar data and ground-truthed feature descriptions, excavation results, radiocarbon chronologies, geoarchaeology and environmental data. Following traditional Samoan protocols, permissions were obtained from the chiefly councils of each village within which research was conducted. All necessary permits for export of research materials were obtained for the described study from the Samoan Ministry of the Prime Minister and Cabinet and the research complied with all relevant regulations. The National University of Samoa Research and Ethics Committee (UREC) approved this research. Given the permission of chiefly councils, individual consents were not required, nor were individuals a subject of research. See S1 Checklist for further information.

### Lidar feature identification

A lidar dataset (S1 Appendix) underpinned our large-scale pedestrian survey and excavation work (see S2 Appendix for acquisition details). Lidar returns cover the first 6–7 km of the valley from sea to inland, but are absent or limited for the upper 3 km of the west branch of the valley and the upper 1.5 km in the east branch of the valley. In total, lidar acquisition allows the investigation of roughly 19 km^2^ of the valley bottom. Importantly, in the project area point densities of different returns varied significantly across the valley and rates of ground returns range from 0 points/m^2^ in areas of dense canopy cover or dense understory vegetation to greater than 10 points/m^2^ in cleared spaces (e.g., roads, cleared farmland), with an average of 0.85 points/m^2^. Areas of lower point densities are correlated with fewer lidar-feature identifications (S1 Fig).

The classified.las datasets were used to create different digital surface models (DSMs) and slope maps and surface archaeological features were identified from these outputs (see S2 Appendix). Though each feature type (i.e., mound/platform, wall, and ditch) displays a distinct set of attributes (see Table 1), individual features are highly variable and, combined with the coarseness of the lidar dataset, it was difficult to develop successful automated or semi-automated feature extraction techniques. Instead, features were digitized manually. Manual digitization revealed areas of the valley that were devoid of archaeological remains. To assess if the lack of lidar-identified features was the result of poor lidar ground return point coverage, the distribution of digitized features was compared to a point density map of ground returns constructed in ArcMap 10.4 (see S1 Fig). Differential ground return density is a well-documented factor affecting feature visibility impacted by the height and density of vegetation across landscapes [76–78].

Given the quality of the lidar dataset and the degree of modern construction in the valley, the goal of lidar feature identification was to examine broad patterns of feature distribution and morphology not feasible through pedestrian survey within typical time and resource constraints of fieldwork. Importantly, we did not attempt to identify all features. The good agreement between ground-truthing and lidar data in the general patterning of features suggests the broad patterns documented by lidar are a valid representation of the archaeological landscape across the valley floor.

### Survey

**Fig 2:**
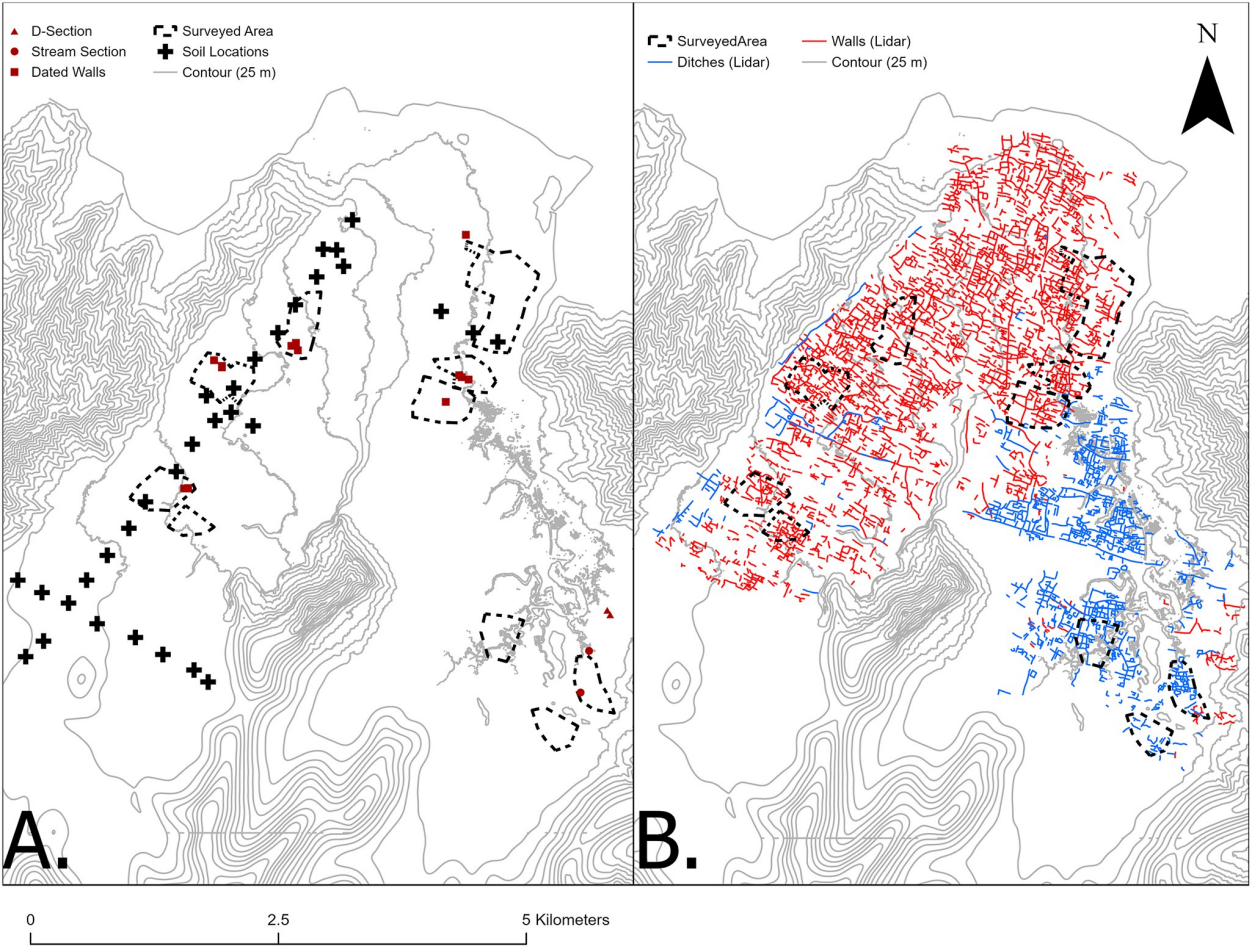
Overview of sampling and survey locations (A), and lidar identified archaeological features (B) in the Falefa Valley.

Systematic pedestrian survey has been carried out in ten locations (Fig 2) to collect feature-scale data such as wall construction variants and ground-truth feature identifications made from the lidar data. Other areas have been surveyed in ad hoc fashion as time permitted when excavating or conducting other work. Systematic surveys were performed by crews of 5–7 people walking transects spaced approximately 5 m apart. All features encountered, whether previously identified via lidar or not, were described following a standardized protocol, photographed, and mapped using GPS devices (Bad Elf GNSS Surveyor) and iPads running the Collector for ArcGIS application. Horizontal precision was typically 1–5 m. Three wall types were identified in the field, generally by variation in width and height and with expectations drawn from previous work [e.g., 69]. These types are not the product of formal classification and we expect to refine types with problem-oriented classifications [sensu 79] in the future.

In addition to systematic pedestrian survey, lidar ground-truthing survey was conducted within specific areas with the goal of assessing the status of each lidar-identified feature: a correctly identified archaeological feature, a probable modern feature, or not a feature. The dimensions of correctly identified archaeological features were modified when necessary (e.g., if only part of a wall was identified via lidar). Within the areas where lidar ground-truthing was conducted each lidar-identified feature was located and assessed, but the total area was not systematically surveyed.

### Excavation

Wall features were excavated to determine variation in their chronology and construction. Excavations focused on triplets, three walls, generally of different types, that were conjoined. This approach allows us to compare the results of radiometric dating and relative dating of conjoined walls. Excavation was conducted by artificial levels (spits) within stratigraphic layers. Excavated matrix (all or a sample) was passed through ¼ inch (6 mm) screens and all artifactual material retained. Special attention was paid to the stratigraphic association of charcoal and internal morphology of the walls. A relatively large screen size was chosen to quickly process matrix, although we recognize that artefacts and charcoal smaller than the mesh was not typically recovered.

### Radiocarbon dating

Radiocarbon dating was conducted on recovered charcoal, with most of these samples identified to the lowest possible taxonomic category to aid selection of short-lived species. Individual dating results are provided in S1 Table and a subset of these were used to construct Bayesian chronological models with OxCal 4.4 [80] (S2 Appendix). The code for these models is in Supporting Information (S1 Code). MCMC results from the Bayesian models were exported in.csv form and further analyzed using ArchaeoPhases [81] in R [R Core 82]. ArchaeoPhases allows for the investigation of joint posterior probabilities and tempo plots were generated to examine the rate of feature construction across the project area [83].

### Geoarchaeology and geochemistry

Multiple locations in the water-logged sediments in the southeastern quadrant of the valley, the Falevao Basin, were investigated with a D-section corer to determine areas with maximum core depths and greatest ease of extraction. Two cores (Falevao 1 and Falevao 19) located 37 m apart (see Fig 2) were chosen for analyses of charcoal and other sediment characteristics. The cores were sectioned into 50 cm segments for removal from the field.

Multiple geoarchaeological and geochemical analyses were conducted on the cores. See S2 Appendix for detailed methods. Both cores were sampled for charcoal at 1cm intervals, and at 4 cm intervals for Total organic carbon/Total nitrogen ratio (TOC/TN), dry bulk density (DBD) and total organic carbon (TOC) and follow Cresswell and Hamilton [84] and Rayment and Lyons [85]. Only one of the cores was analyzed for TOC/N with a random sample from each section of this core (6 total) tested for an inorganic carbonate fraction (all results negative). Single frequency magnetic susceptibility measures were made on both cores following Walden et al. [86]. Seventy samples taken at 10 cm intervals from both cores were used for pXRF analysis with a Bruker Tracer III SD portable X-ray Fluorescence analyzer following procedures given in McAlister and Allen [87] with modifications as noted in S2 Appendix. The top two samples from the cores were not analyzed as these were mostly organic. These same seventy, 10 cm interval samples were also used for laser particle size analysis with a Malvern Mastersizer 3000. The particle size data were split into three percentage categories–clay (<3.5 μm), silt (3.5–63 μm) and sand (>63 μm).

### Soil nutrients

To evaluate variation in underlying soil nutrient levels, 32 composite soil samples were collected from a transect running from the coast to inland on the Mulifanua volcanic series with an additional three transects perpendicular to this (see S2 Appendix for procedures). The northermost perpedicular transect sampled the Lalomaunga Alluvium (see Fig 2). We include here data on soil pH and % Base Saturation as these measures appear correlated to archaeological distributions in Sāmoa [16, 88]. We compared the results of soil testing to elevation and the distribution of archaeological features, largely in dryland areas.

## Results

### Macro-scale feature distribution

**Fig 3:**
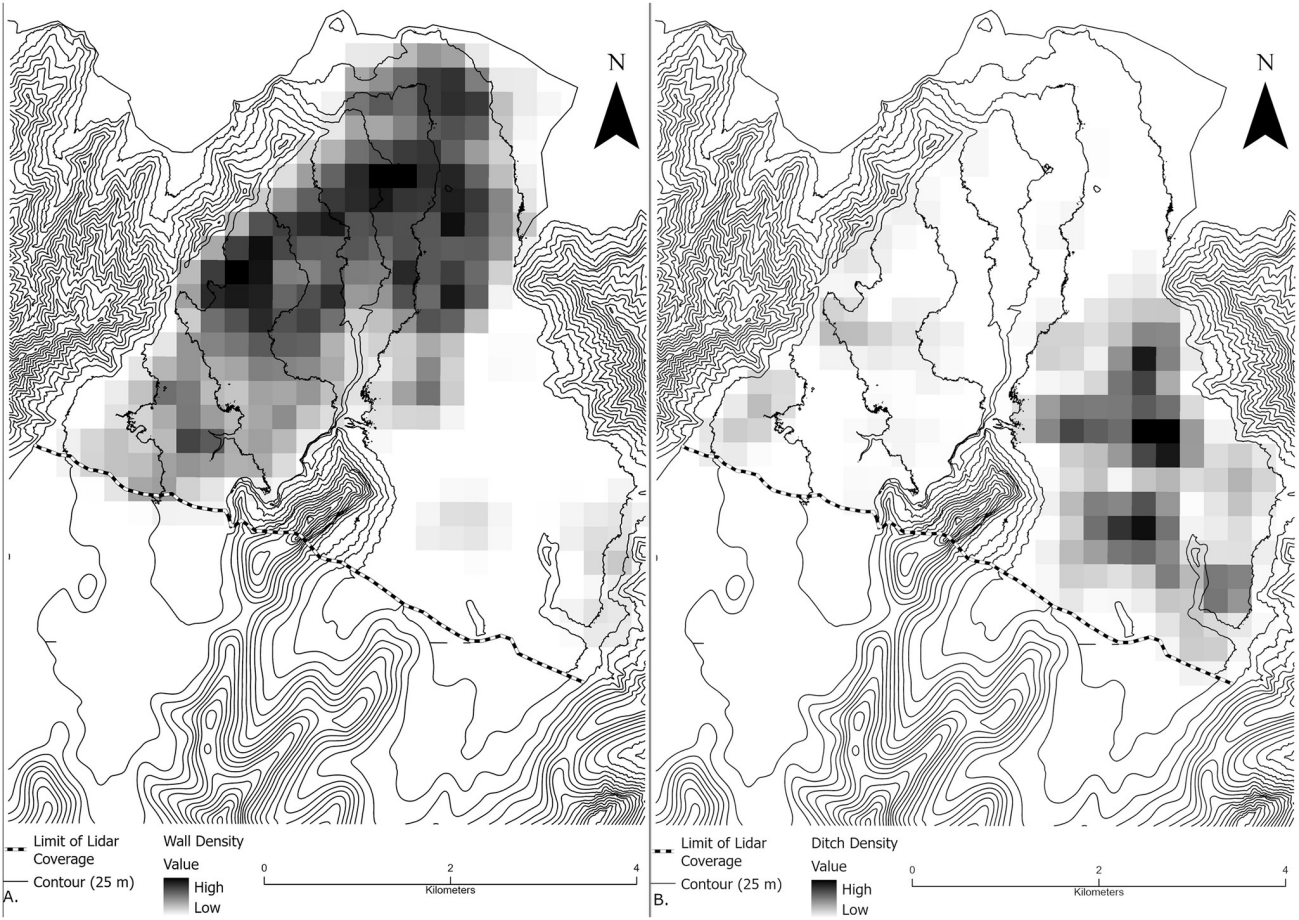
Relative density of walls (A) and ditches (B) recorded digitally. The boundary of the feature distributions toward the inland extent of the valley is tied to the boundary (dashed line) of available ground return lidar data and does not denote the absence of these features. Density was calculated using the line density tool in ArcMap. Cell size is 200 m and density was calculated using a 100 m search radius.

Walls were identified across the entire valley bottom, though their density varies. While there are isolated areas of identified walls in the southeast quadrant of the valley, the highest density is in the northern and western sections (Fig 3A). The area of continuously identified walls is approximately 12 km^2^, which constitutes roughly 54% of the total area of the valley bottom with lidar coverage. Wall lengths are right skewed with a median of 58.8 m (feature data in S3 Appendix). However, some walls were probably much longer in the past as indicated by currently discontinuous wall segments that are roughly positioned along the same line. The three wall types identified in the field include ʻsingle walls’ [following 53], typically about 1 m wide (median 1, IQR 0.75) and a little less than 1 m tall (median 0.73, IQR 0.5). The two other wall types are generally longer (one measures over 1500 m) and include (1) two parallel single walls separated by approximately 2–3 m, conventionally called double-walls or walkways, and (2) walls with a flat, raised center, typically 1 m high (median 1, IQR 0.8), and about 3.5 m wide (median 3.5, IQR 1.1) conventially called raised-walkways or causeways. Collectively we refer to both types as large walls. Quantitative comparisons of wall lengths were not calculated due to breaks in walls that make it difficult to consistently identify a continuous wall segment, but heights and widths between large walls and singles walls are significantly different (Wilcoxon test; height: W = 7122, p-value < 0.00; width: W = 1668, p-value <0.00).

In contrast, identified ditches in Falefa are largely confined to the southeast quadrant of the valley, the Falevao basin, with another smaller cluster of ditching at the western edge of the valley (Fig 3B). Besides these areas of continuous ditching, more isolated examples were also noted in the valley. If these clusters are combined, the area of ditching is at least 4 km^2^, or roughly 21% of the total area under lidar investigation. These systems are morphologically similar to some found in the Rewa Delta, Fiji, where pre-European agriculture included reticulate ditch networks used to grow aroids on the frequently flooded landscape [89]. Further ditching has been observed on the hillslopes of the eastern side of the valley [72]. Like walls, the length of individual ditch segments varies, with most measuring less than 100 m in length (median 63.4, IQR 69.9), but several are in the 400–600 m range. Typical ditch widths are about 2 m (median 2, IQR 1). Typical ditch depths are about half a meter (median 0.4, IQR 0.48).

**Fig 4:**
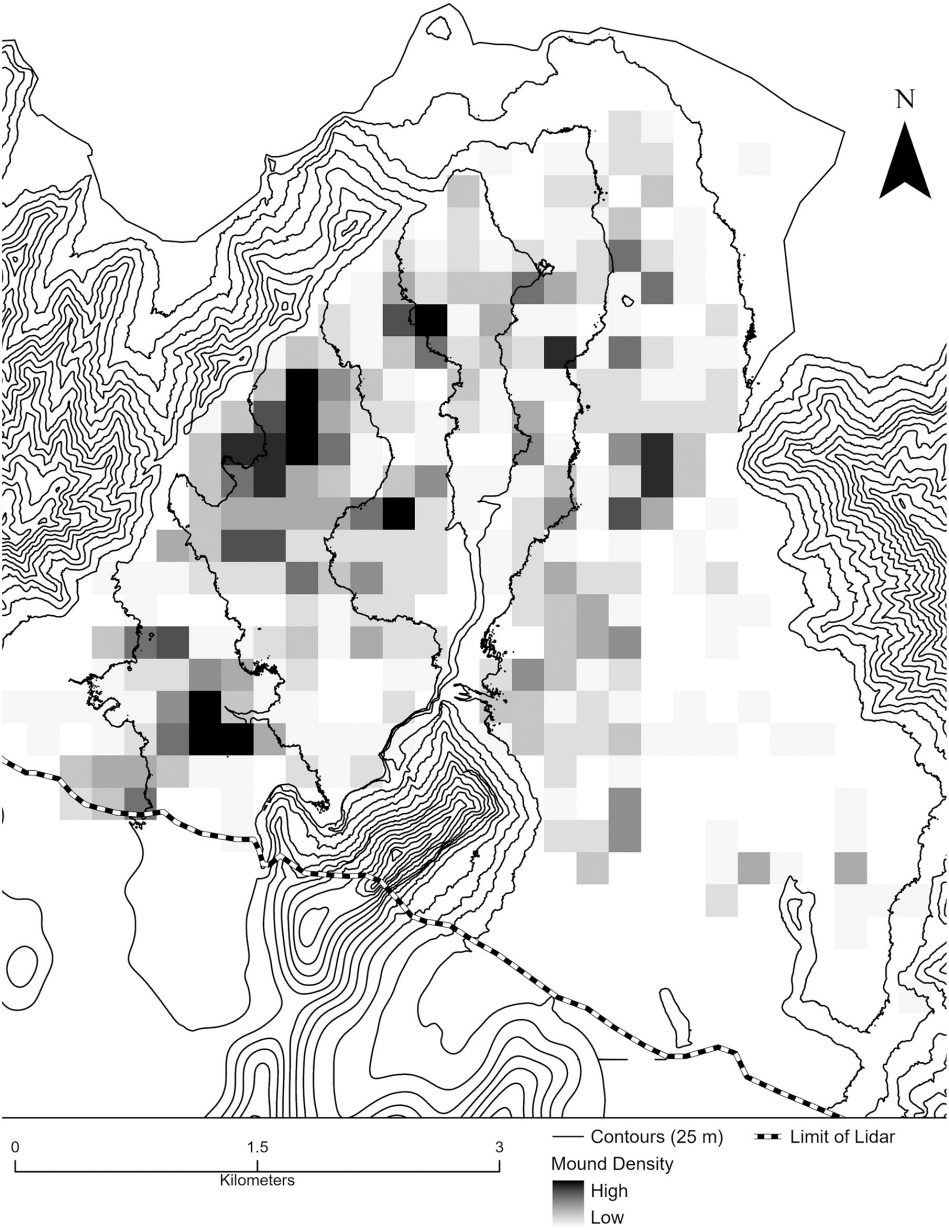
Relative density of mounds recorded digitally. The boundary of the feature distribution toward the inland extent of the valley is tied to the boundary (dashed line) of available ground return lidar data and does not denote the absence of these features. Density was calculated using the line density tool in ArcMap Cell size is 200 m and density was calculated using a 100 m search radius.

**Fig 5:**
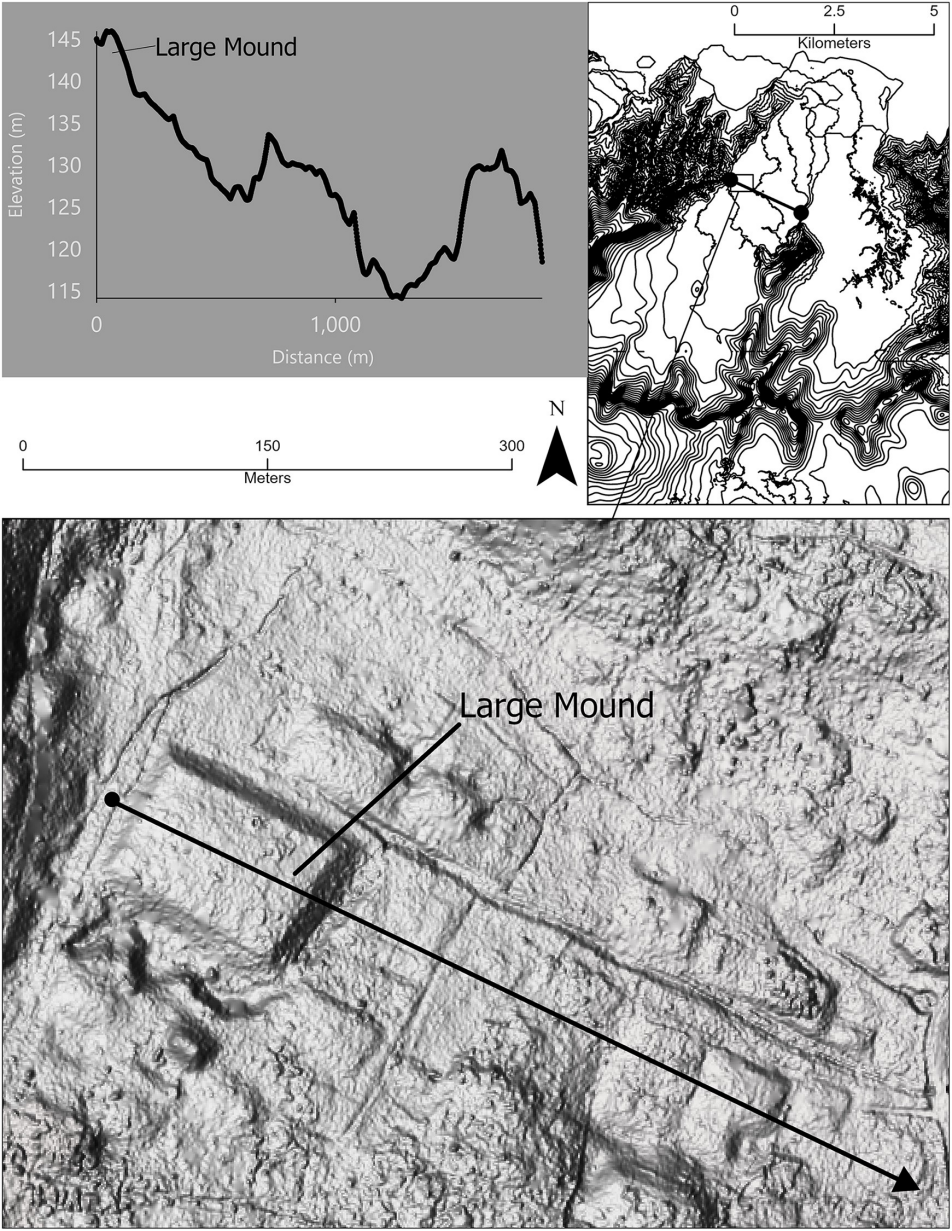
Position of the largest earthen mound in Falefa Valley. The basal dimensions of this feature make it one of the largest earthen features in Sāmoa by that measure. Several additional mounds are visible as well. The location of the western valley profile is indicated by the arrow in the hillshade image and the dot-bounded line in the inset of Falefa Valley. The location of the large mound is indicated by the rectangle in the inset of Falefa Valley.

The distribution of mounds, with roughly rectangular or oval shapes, overlaps with those of ditches and walls, with the greatest density to the north and west (Fig 4). This distribution is biased towards mound features with greater heights as these can be identified digitally. While some mounds were identified in areas of low ground return density, boundaries of these features were difficult to recognize from the lidar data. Given these limitations, as noted above, there is likely an underrepresentation of both lower-height mounds and those in areas of poor ground return coverage. The largest mound in the Falefa Valley (Fig 5), which has a basal area of roughly 13,000 m^2^, is one of the two largest mounds recorded so far in Sāmoa by basal area [90, see also 91]. This is approximately four times larger than the next largest feature in the valley, and, for comparison, larger than the largest *heiau* in the Hawaiian Islands [8:165]. This feature is located prominently within the landscape, especially when viewed from the east. Its position in the landscape along with the presence of borrow pits alongside exaggerates the mound’s size, with the view from the mound extending over half a km to the east and south. Two other large mounds (over 1,300 m^2^) are situated within 300 m with ditching extending east from the area. This complex is located at the far western edge of the valley, roughly equidistant from the back of the valley (6 km) and the coast (4.5 km).

### Micro-scale feature variation

Excavation and pedestrian survey provide a window into micro-scale or local patterning in the Falefa Valley including repeated feature arrangements, wall construction techniques, bonding and abutting patterns of walls, and near-surface sedimentary differences. All features made of stone comprise locally available, undressed, basaltic cobbles and boulders.

**Fig 6:**
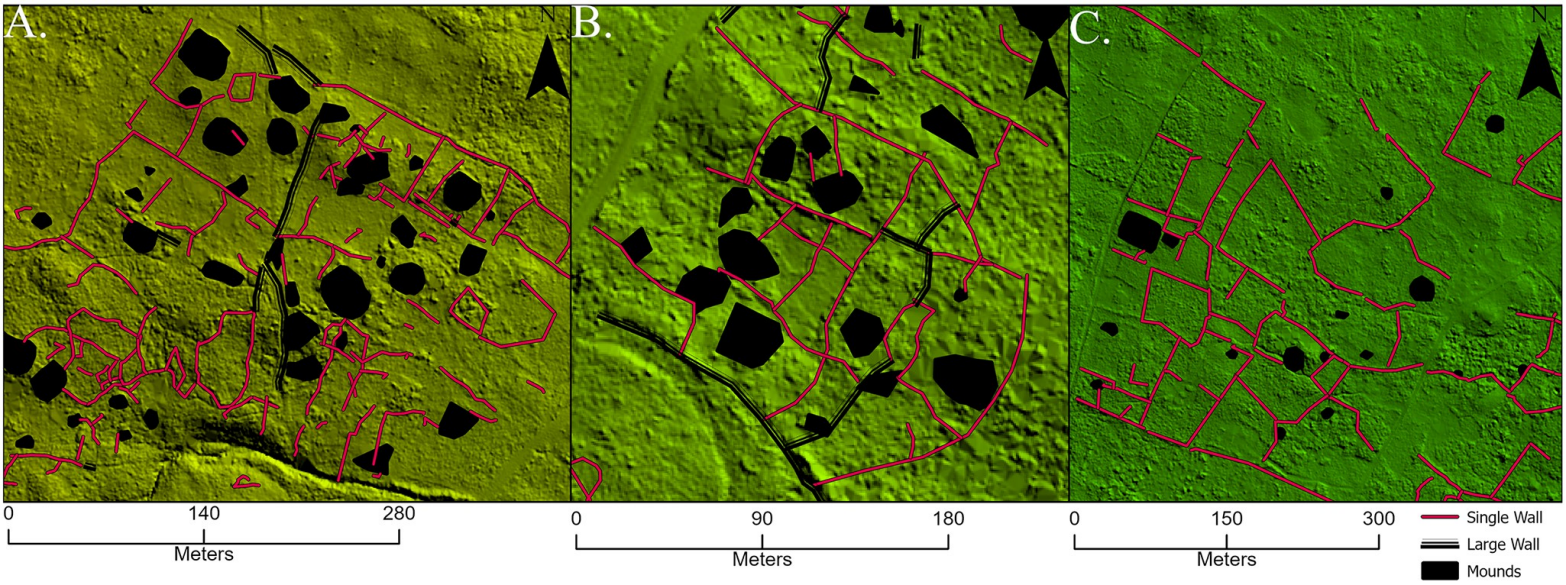
Similar feature arrangements in the Falefa Valley. Walled rectilinear areas often enclose mounds (black) in the southwestern (A), central (B), and (C) eastern portions of the valley, although less often found in the latter. Background is a shaded relief map generated using the lidar dataset with yellow color indicating a higher elevation than green.

Similar feature arrangements of a mound or platform surrounded by a rectilinear perimeter of walls were consistently identified in the field, primarily in the western portion of the valley (Fig 6), similar to Jennings et al.’s [50] HHUs. Our excavations have not yet targeted these locations, so their uses, domestic or otherwise, are currently unclear. Also, their relative prevalence in the western valley compared to elsewhere may result from differential taphonomic processes over space as contemporary households seem to be more widely dispersed inland in the eastern portion of the valley.

Rock walls are core-filled or stacked solid masonry. Core-filled walls have an inner core of randomly arranged (i.e., uncoursed) cobbles surrounded by a veneer of coursed stones on the sides and a top of larger cobbles and boulders. The large walls, including raised walkways, are often core-filled. In contrast, walls made of stacked rocks have continuous masonry courses from face to face and do not have any cross-sectional variation in their construction. All walls are modified by removal and addition of materials over time. These modifications are visible in a number of ways, by noting differences in construction technique and weathering of material along the length of a wall, by noting the removal of wall portions that create discontinuous, but aligned wall sections, and by identifying a linear base course of rocks embedded in the sediment that presumably once supported a wall. This last form of modification is sometimes obvious, with embedded rocks creating a base course that can be 1–3 or more meters wide and stretching for tens of meters or more (S2 Fig).

**Fig 7:**
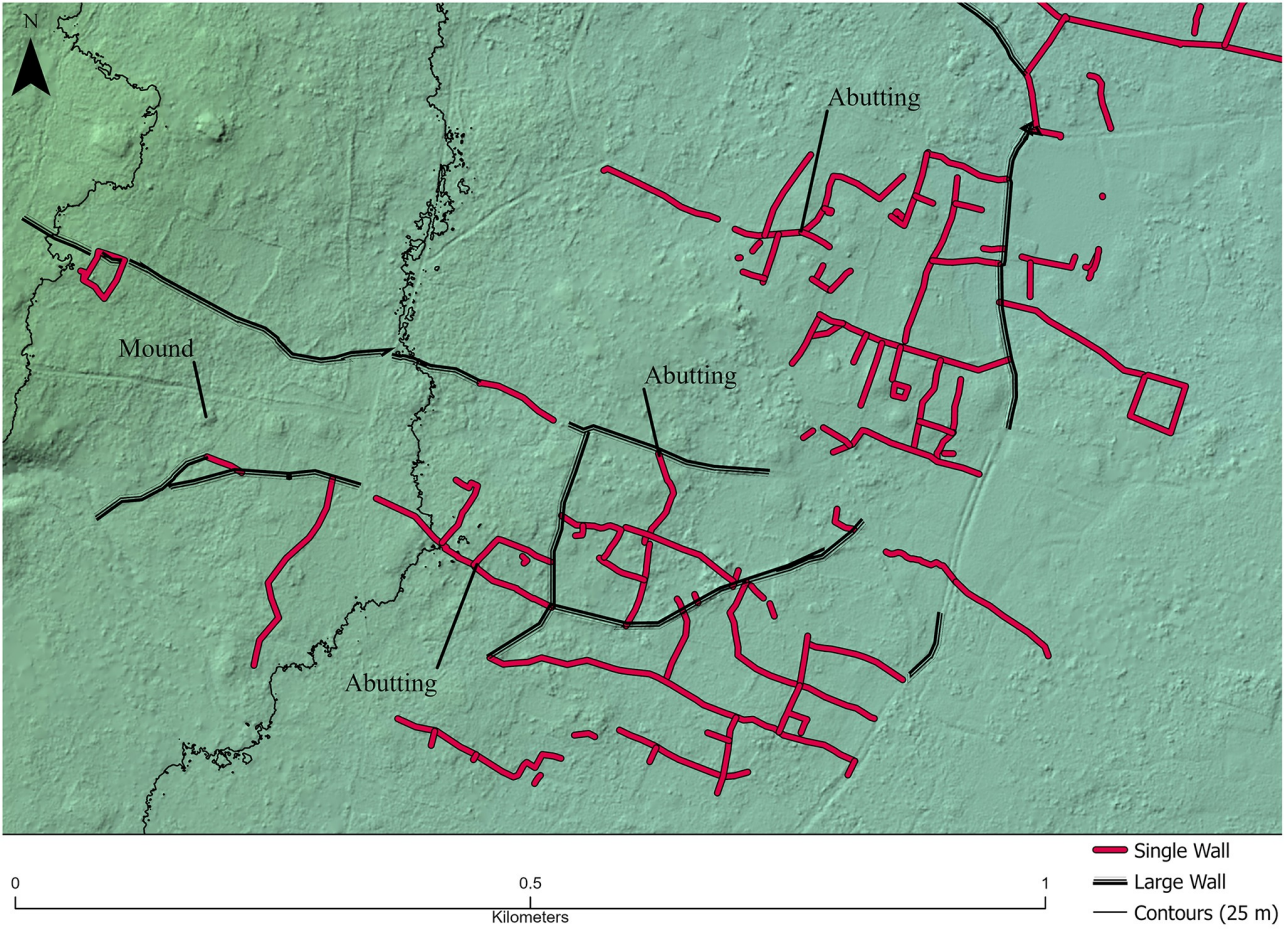
Abutting patterns of walls in the Falefa Valley. Single walls (Red) typically do not cross large walls (Black), abutting them instead, indicating a relative construction chronology. Some single walls also abut each other. Along the length of some large walls, sections may be identified as small walls and this is often the result of stone removal such that the section is less wide or tall and not typical of a large wall. Background in a shaded relief map generated from the lidar dataset (z = 1).

Many walls abut one another or they are bonded with their constituent rocks intermixed. Single walls, for example, rarely cross large walls, instead abutting them and terminating (Fig 7). Such relationships are useful for generating relative wall chronologies and indicate that an abutting wall is typically built after the wall it abuts. These chronological relationships are evidence of land subdivision through time [see 92], although the elapsed time over which this occurs has not been determined.

**Fig 8:**
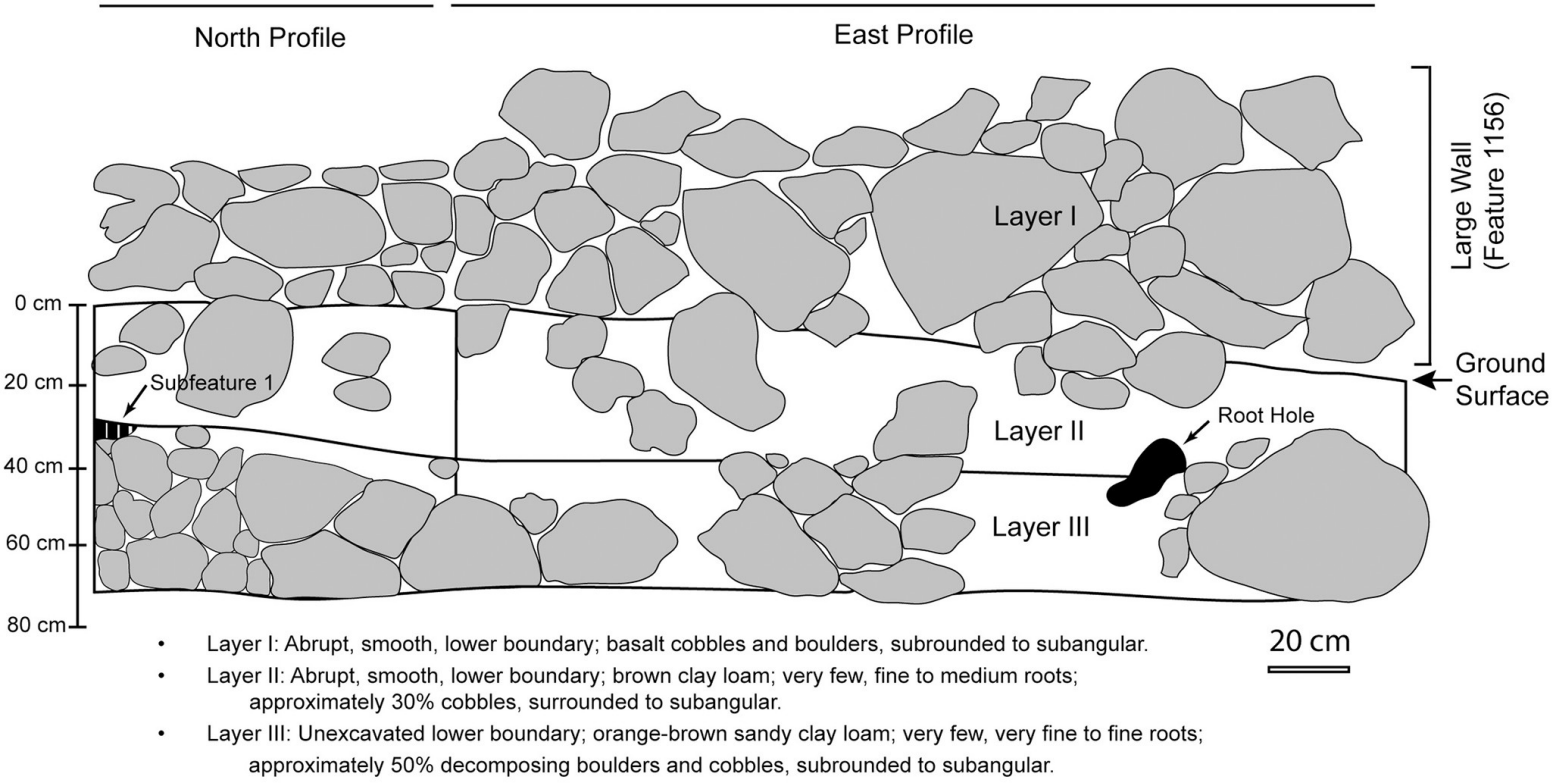
North and east excavation profiles of large wall, feature 1156. Basic layer descriptions are below profile image.

Excavation of wall features revealed a typical stratigraphic sequence (Fig 8). Subrounded to subangular basalt stones forming the features were embedded in an uppermost sediment layer, typically a brown (Munsell colors variable) clay loam or similar structure. Layers below this were often sandier and orange-brown in color (Munsell colors variable). Charcoal chunks and flecking were typically found throughout all layers, occasionally adhering to the underside of feature rocks embedded in sediment. Our interpretation of stratigraphic history, based on field-generated sediment descriptions, is that upper layers with embedded feature rocks are continuously re-worked through natural deposition and erosion, gardening activities, and soil formation. Lower strata, with occasional bedrock boulders, are modified by gardening activities less recently than upper layers, if at all in some cases.

### Chronology of features

Sixty-seven radiocarbon determinations on identified and unidentified wood taxa were generated from samples associated with a landscape burning event and 14 wall features distributed across the study area (see Fig 2). Features are morphologically and chronologically ordered in Table 2, with the oldest events at the top and the most recent at the bottom.

**Table 2:** Modelled ages and calibrated radiocarbon ages for archaeological events in the Falefa Valley.

Feature	Modelled Construction or Event Age (68.3% unless otherwise indicated), BP	Modelled Construction Age (95.4% unless otherwise indicated), BP	Elevation (m)	Substrate ^a^
Large Walls				
1156	906–714	914–243 (95%)	28	LA
4108	616–593, 551–539	624–565, 559–510	141	MV
1164	573–437	583–197	27	LA
6000	503–487	509–469	143	MV
4150	478–311	490–179	26	LA
5052	286–243	293–131	118	MV
5023	273–170	281–89	139	MV
Single Walls				
2084	480–193 (67.7%)	482–79 (86.4%)	145	MV
2086	476–193	480–28 (94.6%)	144	MV
5063	467–277	479–82 (93.7%)	113	MV
3335	288–191, 160–72, 67–63	290–12	25	LA
5058	272–116 (66.9%)	274–44 (86.6%)	118	MV
3330	159–49, 21–4	203–0	26	LA
5021	152–3	239–0	140	MV
Burn Event				
	Calibrated Range (1 s.d.)	Calibrated Range (2 s.d.)		
Stream section 3201	675–656	681–649 (91%), 580–573 (4.4%)		LA
Stream section 3206	675–655	681–650 (92%), 580–573 (3.9%)		LA

^a^ LA, Lalomaunga Alluvium; MV, Mulifanua Volcanics

Our presentation of the feature chronology relies upon the 95.4% HPD and 2 sd calibrated ranges as these are the most accurate results. All features date from the last thousand years, with all but one dating to the last approximately 620 years, although the single exception to this has a modeled construction age with an almost 700-year range. The oldest walls in the chronology are large walls, and all of these are of the raised-walkway type. The oldest large wall (1156) may have been built before an approximately 650 cal BP burn event recorded in two stream sections (3201, 3206, see Table 2) in the southeastern portion of the valley. These sections are 440 m apart and both include a 10–20 cm layer, approximately 1 m below the ground surface, containing abundant charcoal with *Cocos nucifera*, copius (e.g., multiple 1-litre bags) *A*. *moluccana* (candlenut) nut endocarps, and unidentified organic matter. As *A*. *moluccana* endocarp fragments from these separate sections returned the same date range (see S1 Table) we interpret the charcoal- and organic-rich strata from which they derive to record a burn event of modest size, at least large enough to encompass an area greater than 400 m in length. Significantly, the likely Polynesian introduced *A*. *moluccana* is rare or absent from ‘Upolu Island today, nor have we seen any trees in the Falefa Valley.

**Fig 9:**
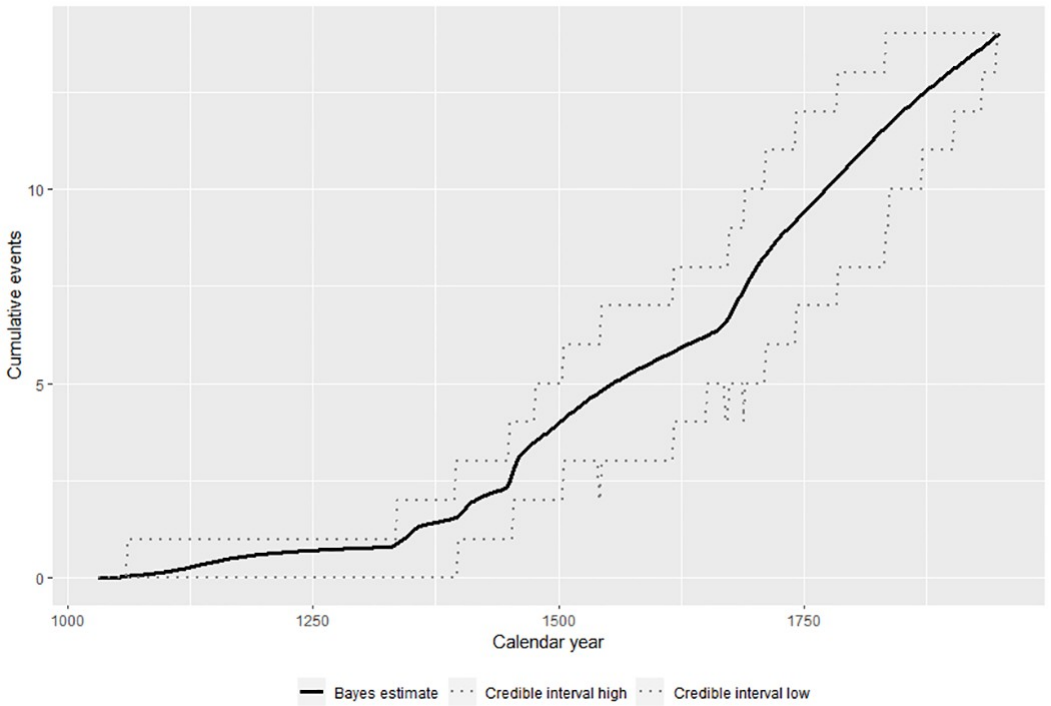
Tempo of wall construction across the Falefa Valley. MCMC results from the Bayesian models were analyzed using ArchaeoPhases [81] in R [R Core 82]. Note the increase in wall construction in the 14^th^ century and later.

After the burn event, additional large walls were constructed in the northwestern and eastern parts of the valley. Single walls may have been built as early as the 15^th^ century, but all are built after the burn event and the construction of large and small walls increases in frequency through time (Fig 9). Finally, the burn event strata, given that they are approximately 1 m below the current ground surface, provide a relative measure of ditch chronology in the immediate area. The mean depth of ditches within 300–400 m of the burn event strata is 21 cm (sd 14 cm) and thus even considering post-use sedimentation within the ditches it seems probable that they were all constructed after the burn event around 650 calBP.

### Paleoenvironmental variation

Datable material from the D-section core Falevao 19 indicates the sedimentary record recovered from Falevao basin begins prior to 5700 cal BP and continues to the present. Three dates (S1 Table) from the core define three temporal phases: pre-human deposits stratigraphically inferior to the range of 5900–5719 cal BP (310–311 cm below surface); pre-human deposits superior to that range and up to anthropogenic deposits dated at 669-629/593-560 cal BP (190 cm); and late anthropogenic deposits dating from this more recent range up to the present. A single sample of datable material from core Falevao 1 corroborates this sequence with an additional date relative to Samoan occupation of 927-901/870-797 cal BP (219–220.5 cm below surface). The analytical results from both cores are similar (core data in S4 Appendix). The results below focus on Falevao19 due to the greater chronological resolution of that core.

**Fig 10:**
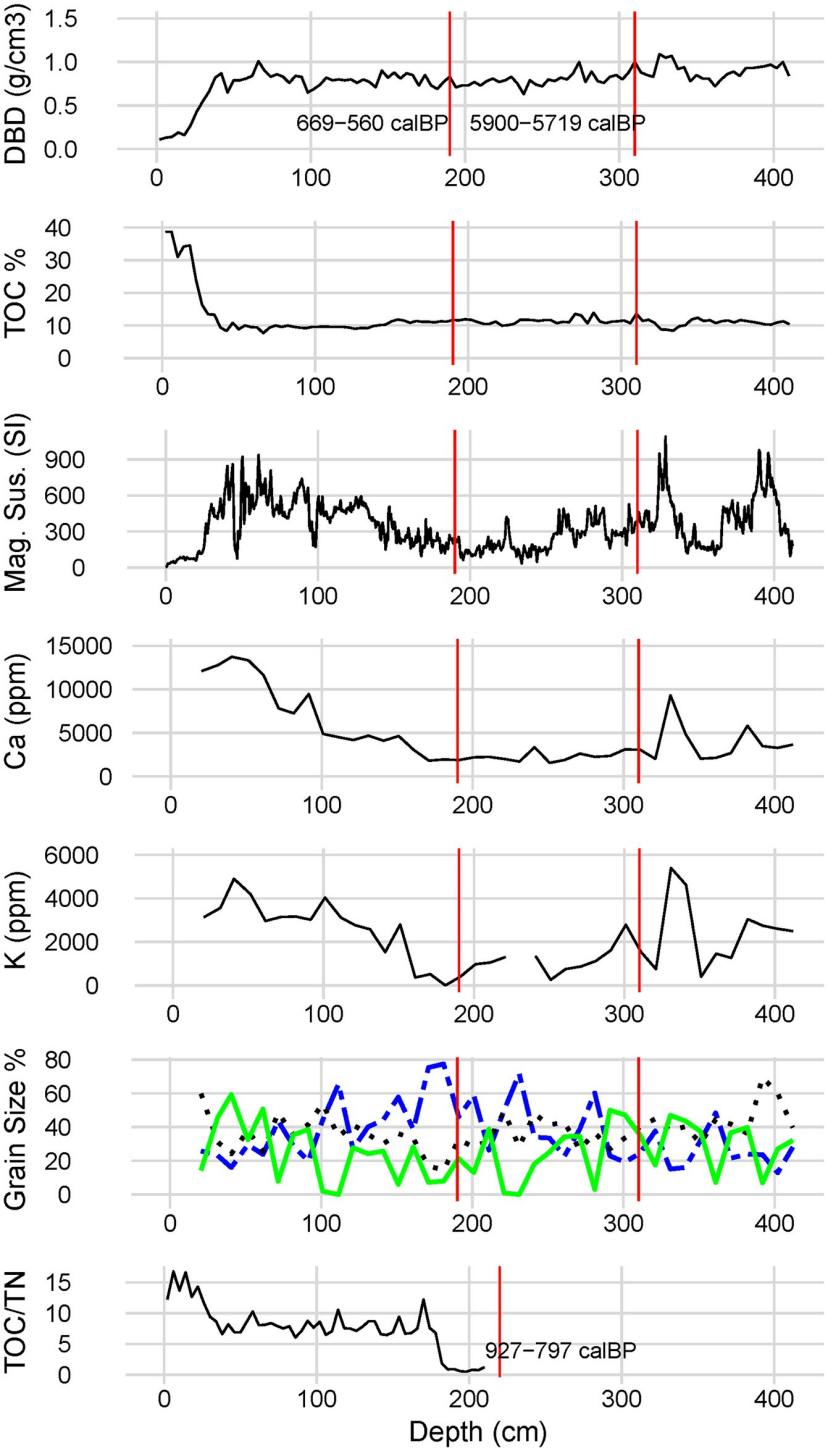
Results of various analyses on D-section cores. Depths are marked along the x axes. Red lines indicate the depths and results for radiocarbon analyses of samples Wk-51320, -51321, and -51322. Bottom graph from analysis of Falevao 1 core. All other results from Falevao 19 core. Missing values for K and TOC/TN result from no samples measured at those depths. Graph of grain size percents: green, solid line is sand, black, dotted line is silt, blue, dashed line is clay.

The Dry Bulk Density (DBD) values trend slightly upward with depth (Fig 10), as is expected by compaction of deeper sediments (van den Akker 2005). Values in the top 26 cm range from 0.11–0.43 g/cm³ (FV19) which is consistent with organic rich, peaty soils. The remaining values to 410 cm range from 0.63–1.09 g/cm³ and are within the range expected for fine silts and clays (Cresswell and Hamilton 2002).

There is no clear increasing or decreasing trend in the TOC data (see Fig 10) except the sharp increase in the top 26 cm comprising topsoil. These uppermost TOC values all fall within a “high” range of TOC consistent with humus rich soils [93].

Magnetic susceptibility values strongly infer depositional changes recorded in core sediments (see Fig 10). These values show the fluctuating wet-dry environment of the Falevao basin, picking up relatively small influxes of eroded sediments and magnetic minerals. The magnetic susceptibility values also record more significant upslope erosional events represented by large peaks in the magnetic susceptibility, including two peaks prior to 5900–5719 cal BP, almost three millennia before humans arrived in Sāmoa. After this time to 669-629/593-560 cal BP, there is a trend of decreasing magnetic susceptibility, although this trend includes significant variation in high and low values. After 669-629/593-560 cal BP there is an increase in magnetic susceptibility values, until these values decline quickly in the upper 26 cm of topsoil. After 669-629/593-560 cal BP, variance in magnetic susceptibility also increases beyond that for any time during human occupation of Sāmoa. Additionally, after this time the concentration of detrital indicators Ca and K increases indicating the weathering of Ca and K bearing minerals into the catchment (see Fig 10).

TOC/TN data were generated for part of the Falevao 1 core (2-210cm). The values for the deepest sediments, very probably sometime before the 650–550 cal BP date in core Falevao 19, are very low (< 2). Beginning at a depth of about 175 cm there is a significant increase in TOC/TN values (ranging from approximately 6–10) indicating a dominantly aquatic source for organic matter and in the top 26 cm the values increase again (ranging from approximately 11–17) indicating mixed aquatic and terrestrial sources. Although there is no date on material from the Falevao 1 core more recent than 927–797 cal BP, the increase in TOC/TN values at approximately 175 cm occurs at the same depth as the increase in both magnetic susceptibility, and K and Ca ppm values, and appears to signal the same depositional event as those measures.

Taken together, the Falevao 1 and 19 core analyses indicate a relatively stable and continuous depositional phase from first human settlement to 669-629/593-560 cal BP. From this time onwards, these data reveal altered human-environment interactions in the Falevao basin. The magnetic susceptibility data shows a complicated cycle of erosional and alluvial or colluvial depositional periods, with an erosional trend increasing until it drops off at the topsoil, approximately 25 cm below the surface. TOC/TN data reflect a relatively large decrease in N% around this same time, likely caused by newly unprotected soils that were increasingly eroded into the basin after partial forest clearance upslope. This is supported by several observations. There is no relative increase in TOC% which remains fairly consistent. The increased TOC/TN is not high enough to suggest new inputs of cleared forest detritus, except in the topsoil zone where values are indicative of organic-rich peat [93]. The particle size data (see Fig 10) corroborates the erosional inferences of the other analyses, with the amount of larger sand sized particles generally increasing after 669-629/593-560 cal BP (except for the topsoil), and becoming the dominate particle size in the deposits.

### Soil nutrient variation

**Fig 11:**
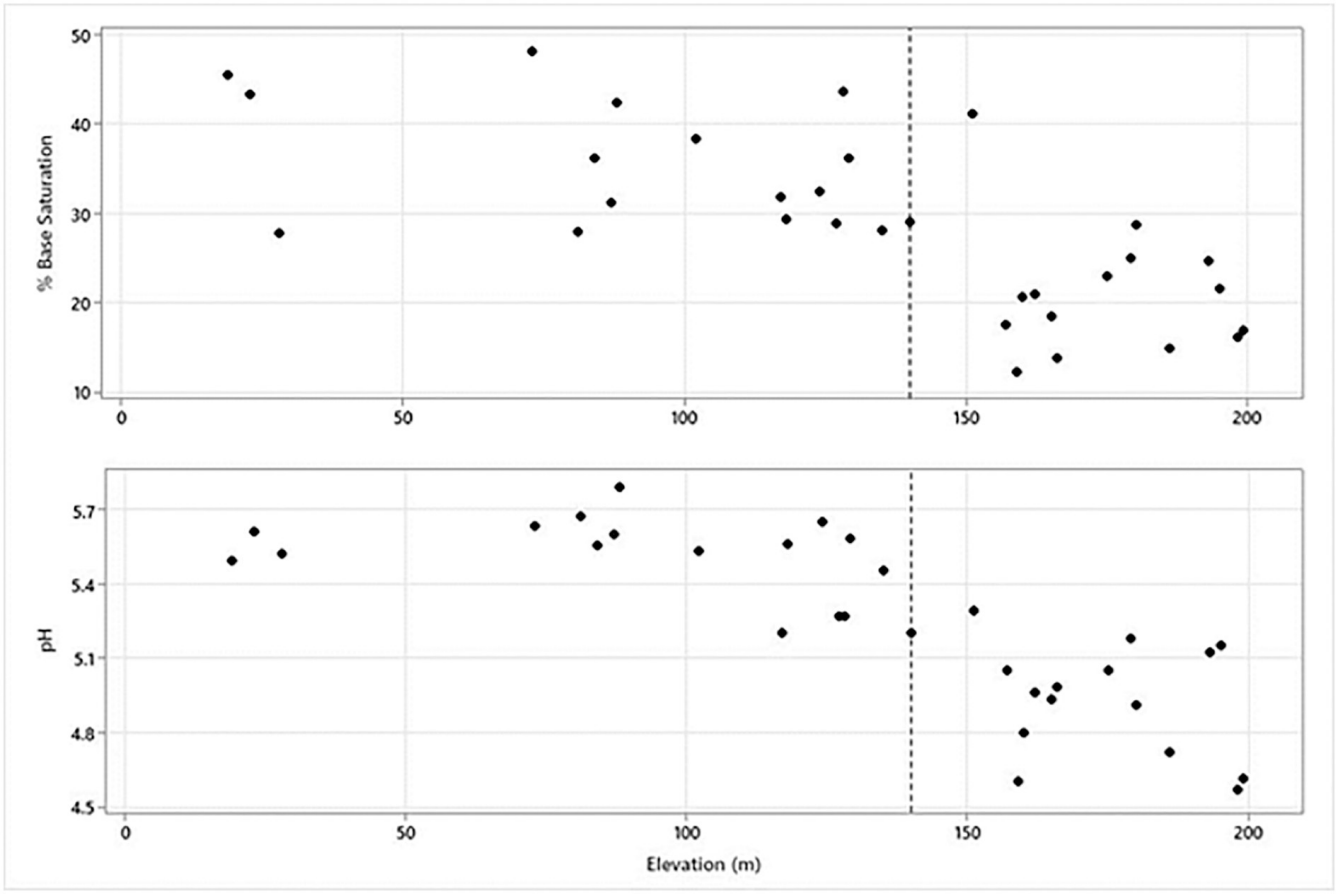
pH and %Base Saturation of Falefa Valley soils. The three samples on the left side of each graph are from the Lalomauga Alluvium in the northeastern portion of the valley. All other samples are from the Mulifanua Volcanics.

Agriculture removes nutrients from the environment and inputs them into edible crops. While there are many elemental and other variables that characterize nutrient availability, both soil pH and % Base Saturation are reliable general indicators for crops on tropical Pacific islands [94, 95], and Sāmoa specifically [88]. Soil pH and % Base Saturation (see S5 Appendix for data) vary considerably through the transects (Fig 11). Soils across the sampled areas are medium to strongly acidic, ranging from 5.7 to 4.5. All values of % Base Saturation are lower than 50%, ranging from 48% to 12%. Each measure, generally, declines with increasing elevation. The decline in pH with increasing elevation is roughly linear on the Mulifanua Volcanic series substrate (coef = -0.008; r2 = 0.70; F = 6.13; p < 0.001). The linear relationship between % Base Saturation and elevation is also significant, but that correlation is not as strong (coef = -0.1819; r2 = 0.53; F = 30.18; p < 0.001). A sharp decline in % Base Saturation values occurs around 150–160 masl, with all samples above 155 masl exhibiting % Base Saturation values below 30%, a threshold constraining dryland agricultural patterning in Polynesia, including in Sāmoa [16, 88, 94]. The large mound complex is located near this elevational boundary. Furthermore, with the caveat that documented wall density is partially a product of lidar data quality, areas of high wall density are all seaward of this elevational boundary on the younger substrate.

## Discussion

The landscape of the Falefa Valley, its ditches, mounds, platforms, rock walls and sediments, comprises an extensively and variably modified environment. While work continues on the archaeological chronology, our results show consistent ages across feature types that indicate large rock walls were constructed early, possibly as early as 800 years ago. The labor to build a large rock wall, some stretching over a kilometer in length, and 3 meters in width, is greater than the labor to build single walls, and was probably organized differently, possibly related to status differences amongst builders and including pooled labor from emically recognized genealogical lineages. Lineages are the most important organizing principle of Sāmoan society [96, 97]. Large rock walls divide (or possibly, connect) the landscape differently than single walls too, with the latter often enclosing smaller areas, and abutting the large walls.

The earliest large rock walls are penecontemporaneous with a sharp population rise across Sāmoa documented by Harris et al. [17]. They identify a small founding population for Sāmoa approximately 3000 years ago and this is corroborated archaeologically [44–46]. Sāmoa’s population remained relatively small, until a sharp rise 900–1050 years ago that eventually tripled the effective population size present at the end of the first millennium [17: Fig 4], a pattern consistent with archaeological data elsewhere in the archipelago [16].

Within a couple hundred years of this population rise, large rock-wall building starts, and shortly thereafter in the Falevao basin of the southeastern valley people burned a landscape of *A*. *moluccana* trees, certainly including other taxa and vegetation types. *A*. *moluccana* is likely not native [60, 98] and its introduction by Sāmoan ancestors indicates the presence of an arboricultural landscape in the valley by at least this time. The removal of *A*. *moluccana* stands is associated with a sequence of silt and clay deposition in this relatively low elevation area, followed by the excavation of reticulated ditch networks. These ditches removed water from the landscape, channeling it to the river and streams [54].

Although large rock walls continued to be built, single walls are built with increasing frequency after approximately 480 years ago (see Table 2). Single walls are shorter than large rock walls and more often enclose areas, sometimes adjacent to large rock walls. Given their smaller size and shorter lengths compared to large rock walls, single walls are probably associated with different behaviours, such as dividing the landscape into farming plots.

Mounds and platforms, both earth and rock, comprise another prominent feature on the landscape and these range from massive constructions equaling some of the largest monumental features in the Pacific, to mounds that might have supported a single small structure, similar to house mounds in Sāmoa today. This variable construction, including occasional rock paving and internal structures like ramps or raised areas, also indicates different labor organization and associated behaviors. Dating analyses of mound construction are not yet complete, but are underway.

All of these features exhibit relatively discrete spatial patterns. Rock walls of both types and mounds are concentrated in the western and northern portions of the valley, while the density of ditches is greatest in the southeastern quadrant. These features are also associated with varying agricultural potential measured by pH and %Base Saturation. On the Mulifanua volcanics, these values decline towards the back of the valley, above approximately 140 m elevation. Rock wall density on this landform is greatest seaward of this point, toward the front of the valley. Ditch density is greatest in the southwest quadrant where water-logged sediments are found. Indeed, ditches are almost solely found in this area.

Our work reveals a series of empirical correlations related to agriculture, land tenure and social change in Sāmoa including population rise and rock wall construction, wall types and chronology, ditching and human-induced environmental change, feature distributions and agricultural fertility. We next propose a set of processes to explain these correlations and describe the empirical work necessary to evaluate these proposals in future research.

### The advent of territoriality

To be explained by directional selection, distinct from, for example, stabilizing selection, territorial behaviors should proliferate in a population at the expense of non-territorial behaviors, at least initially. Additionally, the benefits of territoriality, such as increase in exclusive access to resources, increase in offspring, and potentially intergenerational transmission of these benefits, should exceed the costs of this behavior [12, 99]. Similar to other Pacific Island case studies [see 15], applying this cost-benefit logic to the archaeological record of Falefa Valley assumes agricultural land is the resource of interest, the potential fertility of agricultural land varies with location, and access to higher fertility land can be restricted [e.g., 28, 100]. We propose that these assumptions are met in Falefa and that population rise 900–1050 years ago made territoriality a fitness-enhancing strategy [2, 11] under directional selection.

There are different ways to evaluate the hypothesis that population rise in the Falefa Valley generates directional selection for territoriality. We begin with an empirically tractable option that considers both population and resource distributions. Following Harris et al. [17], there would be a smaller population, and one only slowly increasing in size within the Falefa Valley prior to approximately 1000 years ago. Based on models of populations under little competition for resources [99, 101, 102], we assume that approximately 1000 years ago this population would exhibit an ideal free distribution relative to agricultural fertility or be relatively uniform. This assumption is supported by the observation of only a modest gradient in agricultural fertility in the valley, and the presence of *A*. *moluccana* trees, not a primary food source, that were later removed for more productive agriculture. With rapid population rise after about 1000 years ago the availability of agricultural land per capita would decrease [e.g., 103], thus increasing the relative value of higher-fertility land, and creating a steeper resource gradient, or a resource gradient characterized by substantial thresholds. From this point, the population distribution would increasingly fit an Ideal Despotic Distribution [99]. We see evidence of this in our dataset with the advent of walls, the first archaeological marker of territorial behavior, shortly after Harris et al.’s [17] proposed substantial population growth. We further expect that evidence of territorial behavior will be first present in higher fertility areas. It is in these areas we expect the benefits of excluding access to resources will first be greatest. Such territoriality is also a collective action problem [104] as individuals pay a cost, for example in physical resources and time, to produce a collective good. Territoriality is a collective good as it requires coordination amongst individuals; in this case, the collective good may have been found at the lineage or other corporate group scale rather than a larger political unit. Again, we expect solutions to this collective action problem to be found first in areas of higher agricultural fertility as the payoff for territoriality here will be greater and therefore possible even if there are those who do not contribute to territoriality (defectors in a collective action problem).

**Fig 12:**
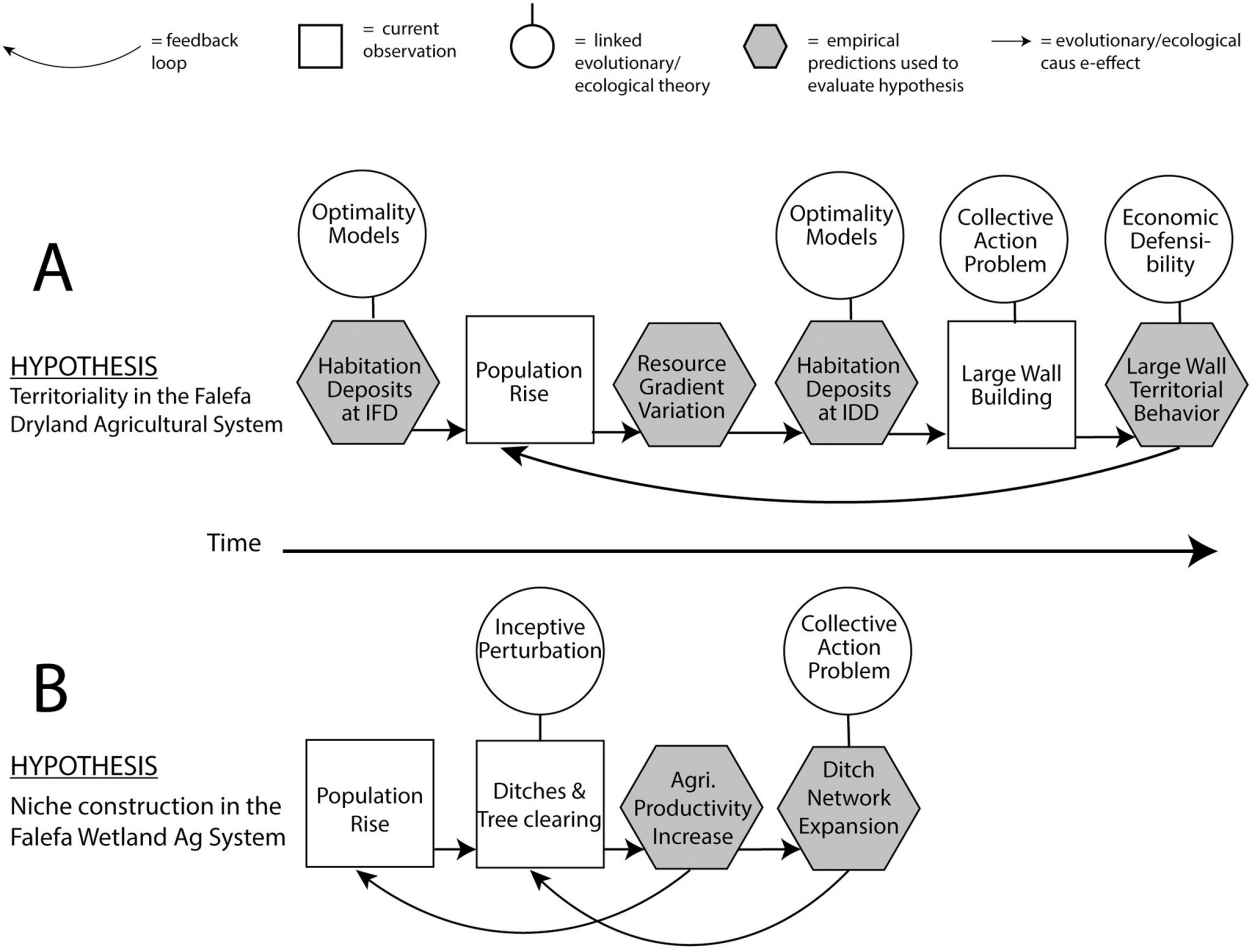
Components of the territoriality and niche construction hypotheses to explain portions of the archaeological record in the Falefa Valley. The diagrams indicate components that can be theoretically evaluated (linked evolutionary/ecological theory), those that are empirical predictions (modelled or hypothesized outcomes) presented here, or components that are current observations.

A schematic representation of this hypothesis focuses attention both on key points where it can be empirically evaluated, and points that are up for theoretical debate (Fig 12). Our hypothesis requires that prior to about 1000 years ago habitation deposits are either relatively uniformly distributed or in accordance with an Ideal Free Distribution in relation to agricultural lands. After about 1000 years ago habitation deposits should increasingly display an Ideal Despotic Distribution associated with areas of higher agricultural fertility. There are currently not enough dated occupation deposits to evaluate this prediction, although similar work by Morrison et al. [105] on Tutuila Island demonstrates the procedures for comparing variable habitats and the distribution of dated deposits in these habitats against IFD expectations.

Additionally, we expect the oldest large walls, the proposed results of territorial behavior, to be located in areas of higher agricultural fertility and to post-date population rise. The oldest large walls (e.g., 1156, 1164, 4108) are indeed in areas of relatively high fertility, below approximately 140 m elevation, although they are built on different substrates and two of the walls (1156 and 1164) are not very near soil fertility sample locations. To be a robust test, the wall chronology should include many more features, and our soil fertility samples should include greater spatial coverage, both a focus of future work.

We see territoriality as the best current explanation of the general changes in the Falefa valley after about 1000 years ago as territoriality encompasses relevant empirical observations and reasonable predictions. The territoriality hypothesis can be challenged on theoretical grounds. In our view, a better hypothesis would include behavioral models that accounted for our current observations and expectations more precisely or that include additional relevant observations and expectations, such as those related to variation in social hierarchies or agricultural intensification, or both.

### Niche construction and new agricultural behaviors

Landscapes are not static and changes to landscapes can modify the costs and benefits of their use. These are central tenets of niche construction theory, wherein environmental modifications by organisms can alter selective conditions causing cultural evolutionary and biological evolutionary change in descendant populations [106]. As agricultural modifications are accretionary, past agricultural behaviors can create the context for future agricultural practices [107]. Agriculture, and the changing productivity of environments over time, are useful examples of niche construction [30].

The landscape of Falefa is a dynamic human-natural system, especially in the southeastern quadrant of the valley, as evidenced by our coring results. This provides us a context for using niche construction concepts to evaluate changes in agricultural behaviors in that location. Of note, these behaviors included the removal of *A*. *moluccana* trees and subsequent depositional changes beginning approximately 600 years ago, both followed by ditch construction. People in the valley would have had an intimate knowledge of the environment, including the effects of forest removal over the previous 2000 years in Samoa [59]. Therefore, we classify forest removal and ditch construction to be a single inceptive behavior that created a new cultivation environment. Variable levels of forest removal on the hillslopes around the southeastern portion of the valley would have had predictable effects, notably, at a high enough level, increased alluvial erosion of sediments and their deposition into the Falevao basin, and like elsewhere in Oceania [108, 109], this would have resulted in marsh covered floodplains highly conducive to wetland cultivation, and replacing a landscape more prone to ponding as suggested by TOC/TNdata from the D-section core.

**Fig 13:**
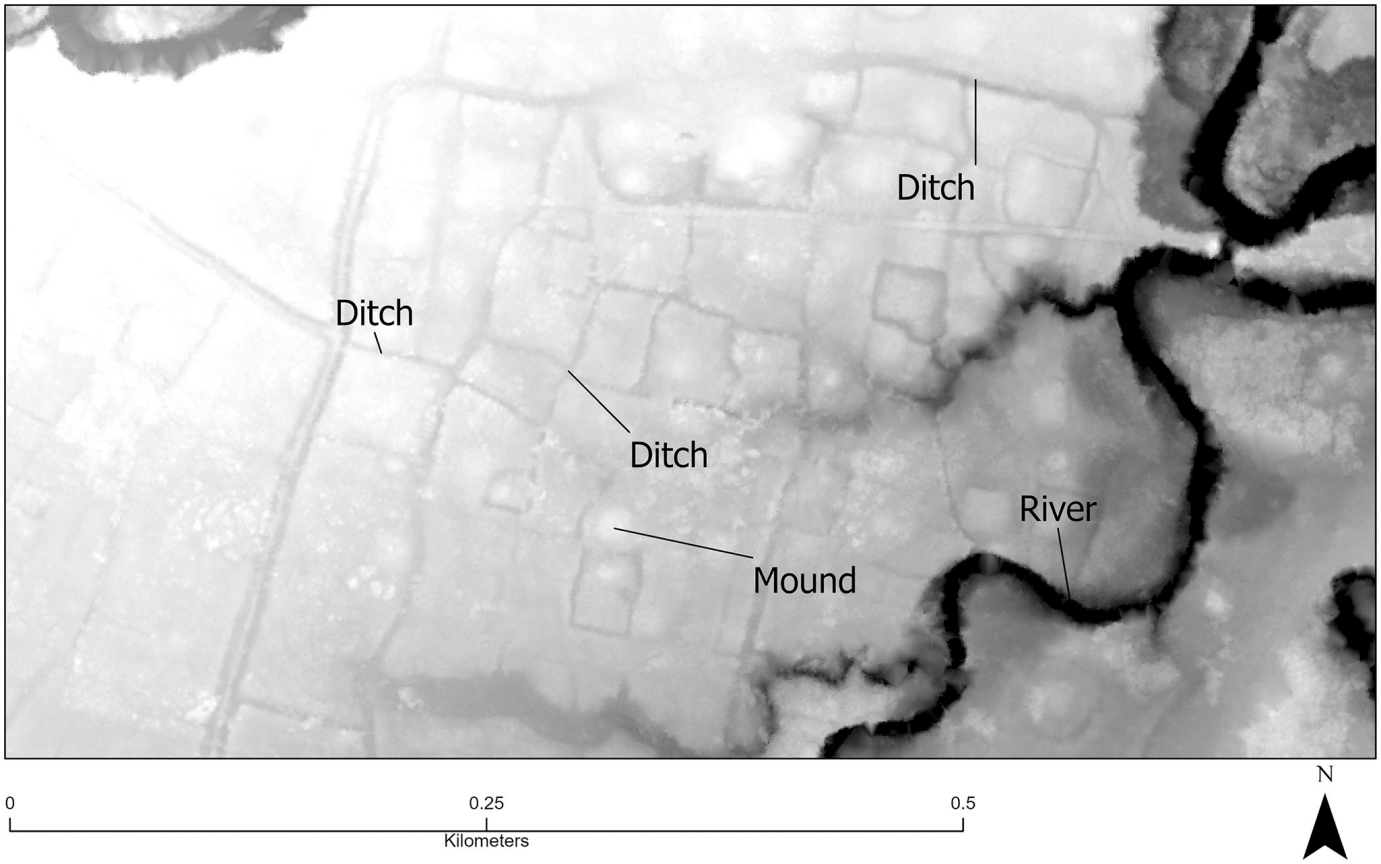
Relative digital surface model of the southeastern Falefa Valley. Elevevation shading is relative to river in black. Reticulated ditches with variable upstream-downstream relationships are shown. Rectilinear light gray areas are mounds.

Given the chronology of environmental change, these behaviors most probably originate after population rise 1000 years ago and we propose that population rise modified the costs and benefits of large-scale environmental modification, including the reduction of *A*. *moluccana* forests. *A*. *moluccana* is an economically useful tree, and one that supports long-term shifting cultivation [110], but it not a primary food resource. The reduction or elimination of *A*. *moluccana* stands (we have seen no *Aleurites* in the valley today), combined with the enhancement of wetland environments through deposition, would have provided opportunities for the expansion of *Colocasia esculenta* (grown in the valley today) or other wetland crop cultivation. Such a change would have altered selective conditions and promoted new kinds of agricultural behaviors, namely investments in drainage ditches. The inundated and managed sediments of the southeastern quadrant would have increased the soil fertility and productive capacity of this area for cultivated aroids. In short, we propose that the southeast quadrant of the valley was transformed into an area of intensified agriculture and the construction and maintenance of ditches would also have presented a new collective action problem as individuals upstream and downstream (Fig 13) must coordinate their construction and maintenance behaviors. The spatial extent of the reticulated ditch network in the southeast also suggests labor coordination at scale similar to that of large-rock walls.

Finally, we hypothesize that these behaviors would have increased within a feedback loop linking greater agricultural productivity and higher population (as identified by [17]), as well as stabilizing or increasing the selective value of cooperative maintenance of ditches to drain agricultural sediments. In turn, this would cascade to impact social systems as production increased, populations became more reliant on increased production, and systems maintenance in a high depositional environment required more labor as the ditch network expanded. Like our territoriality explanation, we can diagram the current empirical observations, empirical predictions, and relevant theoretical concepts (see Fig 12). The chronology of ditching and forest reduction conforms to our expectations of inceptive perturbation [111] and we expect that further dating work on the ditch system will identify its spatial expansion over time. This newly created environment would have increased agricultural productivity (intensification) or perhaps lessened variability, or both. Agricultural intensification and lessened variability may lead to further population rise, perhaps detectable in the radiocarbon record, and create a positive feedback for further inceptive or counteractive perturbation.

## Conclusions

The juxtaposition of large wetland and dryland agricultural systems in a single valley is unusual in Pacific Island societies (indeed, we cannot think of another similar instance). In the Pacific Islands, agricultural systems that incorporate various production strategies are predominantly found in environments with multiple risks [112]. Therefore, we might consider Falefa Valley agriculture to be adapted to different risks linked to acute (e.g., cyclones) and longer term (e.g., decreased rain) climate events, both of which have probably affected Sāmoa for millennia [113, 114]. These different agricultural systems also might create different routes to increased social hierarchy through collective action problems [cf. 115].

Our research has yet to thoroughly investigate the ubiquitous mounds and platforms in the valley. We expect variation in these features, including differences in construction, size, distribution, chronology, and use to be relevant to our hypotheses concerning agriculture and social change. In particular the monumental size of some of these features suggests social hierarchies or a minority of individuals commanding labor within the population, itself a characteristic that can facilitate the collective action problems we note above [19, 116, 117]. We predict that much evidence for increasing social hierarchies will be fairly recent, post-dating the markers of territoriality and inceptive perturbation in the Falefa Valley.

This prediction places increased social hierarchy in the Falefa Valley, and possibly other areas of Sāmoa, after first settlement of East Polynesia and perhaps contemporaneous with the development of increasingly hierarchical social systems there. Thus, we suggest that the rise of social hierarchies in East Polynesian societies is explained by selection working within new niches defined by different resource potentials, environments, and human demographic characteristics [118, 119] and less so by shared ancestry between East and West Polynesia. In short, West Polynesia, or at least Sāmoa, may not be the birthplace of the wider Polynesian chiefdoms [120].

## Supporting information

S1 Checklist Inclusivity in global research. (PDF)

S1 Appendix Statement on lidar data availability. (PDF)

S2 Appendix Additional methods description. (PDF)

S3 Appendix Metric feature data. (PDF)

S4 Appendix D-sections analytical data. (PDF)

S5 Appendix Soil pH and % base saturation data. (PDF)

S1 Fig Comparison of lidar identified features and density of ground returns. (PDF)

S2 Fig Base course of rock wall (Feature 4150). (PDF)

S1 Table Radiocarbon data for all samples. (PDF)

S1 Code Oxcal code for Bayesian chronologiacl models incorporating stratigraphically determined relative constuction order. (DOCX)
