# Supplementary material for: Collective action problems led to the cultural transformation of Sāmoa 800 years ago
Source: PLoS One. 2024 Jun 20;19(6):e0304850. doi: 10.1371/journal.pone.0304850 (PMC11189243; doi:10.1371/journal.pone.0304850)
Supplement: S1 Appendix — (PDF) [file pone.0304850.s002.pdf]

S1 Appendix. Statement on Lidar data availability

The corresponding author obtained the lidar data from The Samoan Ministry of Natural Resources and Environment (MNRE) through an exclusive license stipulating that the data may be used for research only. No special priviledges were given to the authors regarding these data. The lidar data may be obtained from the MNRE by contacting their Spatial Information Agency – Mapping Section through the ministry website: https://www.mnre.gov.ws/
