# Supplementary material for: Collective action problems led to the cultural transformation of Sāmoa 800 years ago
Source: PLoS One. 2024 Jun 20;19(6):e0304850. doi: 10.1371/journal.pone.0304850 (PMC11189243; doi:10.1371/journal.pone.0304850)
Supplement: S2 Appendix — (PDF) [file pone.0304850.s003.pdf]

S2 Appendix. Additional Methods Description

Lidar Feature Identification

Lidar data collection was conducted by Fugro Geospatial Services using a RIEGL LMS-Q780 lidar system fitted onto an AusJet Cessna 441. The survey occurred at a height of 650 m and a maximum speed of 130-140 knots. Data collection used a laser rate of 350 KHz and a line spacing of 423 m, with a minimum pulse density of 4/m2. Some data gaps exist at high elevations due to cloud cover and flying difficulty.

Digital surface models (DSMs) were constructed from classified .las datasets. As some surface features tended to be low to the ground or narrow, a DSM was created to exaggerate vertical and horizontal differences by multiplying elevation (z) values by 3 and reducing the DSM cell output to 0.75 m from the conventional 1 m. Visualization was facilitated by the construction of a series of six hillshades (with azimuths of 20, 90, 160, 230, 300, and 340 and altitudes of 35). Principle component analysis was undertaken on these hillshades to produce a single image, as such PCA images have proven effective for feature identification elsewhere in the Pacific islands [e.g., 1]. Slope maps were generated from the DSM as a complimentary means of identification and visualized as a stretched image using a percent clip function. Finally, a relative DSM was created using the primary road on the western side of the valley and the primary stream on the eastern side of the valley as the constant value. This enhanced the visibility of shallow ditches and low walls. Once features were identified, densities of each feature class were calculated using point, line, and kernel density algorithms in ArcMap 10.4.

Though each feature class (i.e., mound/platform, wall, and ditch) displayed a distinct set of attributes (see Table 1), individual features are highly variable and, combined with the coarseness of the lidar dataset, it was difficult to develop successful automated or semi-

automated feature extraction techniques. Instead, features were digitized manually using the combination of visualizations described above [see 2].

Radiocarbon Dating

Charcoal and organic samples were submitted to either Beta Analytic Incorporated or the Waikato Radiocarbon dating laboratory for analyses.

Radiocarbon sample selection for building OxCal models prioritized samples that would provide the most preicse models for events of interest. Therefore, not all dated samples from a particular feature ares used in model construction.

The date range for the burn layer exposed in stream sections 3201 and 3206 was calibrated using *A. moluccana* endocarp radiocarbon data. In both strata with abundant *A. moluccana* charcoal and other evidence of fire (e.g., orange oxidized sediment) there are also *S. ghaeri* seeds. *S. ghaeri* seeds were dated and returned younger ages by 151 (Wk-48232) and 91 (Wk-48235) conventional radiocarbon years when compared to *A. moluccana* from the same context. However, we use the *A. moluccana* dating analyses to determine the timing of the burn layer for three reasons: (1) it is more likely that the smaller *S. ghaeri* seeds have migrated down the sediment column; (2) we are interested in the removal of the *A. moluccana* trees and therefore directly dated the endocarps; and (3) two *S. ghaeri* seeds returned inverted ages (Wk-48236 and Wk-48235) for their stratigraphic relationship.

Geoarchaeology and Geochemistry

Several analyses were conducted on sediments extracted in two D-setion cores, mostly as 1cm core slices. Additional analytical procedures are described here, in addition to the main text.

Dry Bulk Density (g/cm^3) was calculated by weighing the field-wet sample, then baking at 105°C for two hours after which dry weight was taken and applied to the formula: dry soil weight (g) / soil volume (cm^3) [3]. Total Organic Carbon (TOC) measures were taken by weight before and after baking at 550°C for four hours.

One core was analyzed for TOC/TN ratios following procedures in Rayment and Lyons (2011), with samples from the core oven dried at 40C and ground to homogenize the sediment. A random sample from each section of this core (6 total) was tested for an inorganic carbonate fraction by adding 10% hydrochloric acid (HCl) to a small amount of the sediment, and left overnight. These all returned negative results with no effervescent reactions. Five milligrams of each sample were analyzed in a CNS Elementar, with 5mg standards of sulfanilamide inserted every 15 sediment samples. The values for %TOC and %TN were used to calculate TOC/TN = %TOC/%TN (Rayment and Lyons 2011), providing the weight (as opposed to atomic) ratio of each.

Magnetic susceptibility measures were done using a Bartington magnetic susceptibility meter Model MS2 that oscillates a single frequency through the core, calibrated to take measurements at contiguous 0.5cm intervals [4].

Samples were taken every 10cm as 1cm core slices for pXRF analysis (70 total). These were freeze dried for 48 hours to dry and homogenize the sediment, and thus safe from distortion due to water which can reduce elemental concentrations and skew proportional distributions [e.g., 5]. They were analyzed using a Bruker Tracer III SD portable X-ray Fluorescence (pXRF) analyzer. The instrument employs an X-ray tube with a Rh target and a $10mm^2$ silicon drift detector (SDD) with a typical resolution of 145 eV at 100,000 cps. For this analysis, the X-ray tube was operated with a setting of 40 keV at 25.0μA, through a window composed of 12mil Al

and 1mil Ti filters (Bruker's Yellow filter), giving total element concentrations from K-Th. The top two samples from FV19 were not analyzed as these were mostly organic.

The same 10cm interval samples were also used for particle size analysis after the work on pXRF. For particle size analysis, the samples were covered to ~1cm depth with 10% hydrogen peroxide (H_2O_2), mixed with a vortex mixer, and left overnight to digest any organic matter in the samples. This process was then repeated. Samples were washed with distilled water and centrifuged, and the supernatant fluid poured off. This was repeated three times to remove all H_2O_2. Each sample was run through a 1.4mm sieve to remove larger particles. 0.5% sodium hexametaphosphate ($Na_6[(PO_3)_6]$) was added and the samples vortexed again to disperse the sediment particles. They were then processed using a Malvern Mastersizer 3000. The particle size data was split into three percentage categories – clay (<3.5 μm), silt (3.5-63 μm) and sand (>63 μm).

Soil Nutrients

To evaluate variation in underlying soil nutrient levels, 32 compositive soil samples were collected from a transect running from the coast to inland on the Mulifanua volcanic series with an additional three transects perpendicular to this. The northermost perpedicular transect sampled the Lalomaunga Alluvium. Each composite sample represents three independent sampling locations in proximity (< 20 m), integrating soils 0-30 cm depth below the surface, following methods in Vitousek et al. [6]. We included here data on soil pH and % Base Saturation as these measures appear correlated to archaeological distributions in Sāmoa [7, 8]. All soil samples were air-dried and sieved through 2 mm mesh. Soil pH was measured on air-dried soil mixed with deionized water at a 1:2 ratio. Base Saturation was measured using the ammonium acetate

(NH4OAc) method buffered at pH 7 following procedures in the Soil Survey Manual [9]. We compared the results of soil testing to elevation and the distribution of archaeological features, largely in dryland areas.

References

1. Jones BD, Ladefoged TN, Asner G. Tracing the resilience and revitalisation of historic taro production in the Waipi‘o Valley, Hawai‘i. The Journal of the Polynesian Society. 2015;124(1):83-109.

2. Quintus S, Day SS, Smith NJ. The Efficacy and Analytical Importance of Manual Feature Extraction Using Lidar Datasets. Advances in Archaeological Practice. 2017;5(4):351-64. Epub 2017/08/23. doi: 10.1017/aap.2017.13.

3. Cresswell H, Hamilton G. Bulk density and pore space relations. Soil physical measurement and interpretation for land evaluation. 2002:35-58.

4. Gale S, Hoare PG. Quaternary sediments: petrographic methods for the study of unlithified rocks: Blackburn Press; 2012.

5. Williams R, Taylor G, Orr C. pXRF method development for elemental analysis of archaeological soil. Archaeometry. 2020;62(6):1145-63.

6. Vitousek PM, Ladefoged T, Hartshorn A, Kirch PV, Graves M. Soils, agriculture, and society in precontact Hawai'i. Science. 2004;304:1665.

7. Autufuga D, Quintus S, Yoo K, Day S, Huebert J, Deenik J, et al. Distribution of Soil Nutrients and Ancient Agriculture on Young Volcanic Soils of Ta‘ū, American Samoa. Soil Systems. 2023;7(2):52. PubMed PMID: doi:10.3390/soilsystems7020052.

8. Quintus S, Autufuga D, Day S, Huebert J, Lincoln NK, Motu N, et al. Tracking Emergent Spatial and Social Patterns across Terraced Landscapes in Polynesia. Journal of Field Archaeology. 2022;47(3):196-211. doi: 10.1080/00934690.2021.2018259.

9. Staff SSD. Soil survey manual. In: Agriculture UDo, editor. Washington, D.C.: Government Printing Office; 2017.
