# Supplementary material for: Collective action problems led to the cultural transformation of Sāmoa 800 years ago
Source: PLoS One. 2024 Jun 20;19(6):e0304850. doi: 10.1371/journal.pone.0304850 (PMC11189243; doi:10.1371/journal.pone.0304850)
Supplement: S3 Appendix — (PDF) [file pone.0304850.s004.pdf]

S3. Appendix. Metric Feature Data

Metric measurements of walls and ditches and an R script to calculate reported values are available at 10.17608/k6.auckland.25058162. Precise wall and ditch locations are not included as consent to release these data has not been given by invididual land-holders.
