# Supplementary material for: Collective action problems led to the cultural transformation of Sāmoa 800 years ago
Source: PLoS One. 2024 Jun 20;19(6):e0304850. doi: 10.1371/journal.pone.0304850 (PMC11189243; doi:10.1371/journal.pone.0304850)
Supplement: S4 Appendix — (PDF) [file pone.0304850.s005.pdf]

S4. Appendix D-Sections Analytical Data

Analytical results of the D-section core analyses and an R script to calculate reported values are available at 10.17608/k6.auckland.25142762
