# Supplementary material for: Collective action problems led to the cultural transformation of Sāmoa 800 years ago
Source: PLoS One. 2024 Jun 20;19(6):e0304850. doi: 10.1371/journal.pone.0304850 (PMC11189243; doi:10.1371/journal.pone.0304850)
Supplement: S5 Appendix — (PDF) [file pone.0304850.s006.pdf]

S5. Appendix. Soil pH and % Base Saturation Data

Raw data, pH, and % Base Saturation data for soil samples used in the analyses are available at 10.17608/k6.auckland.25844494
