# Supplementary material for: Collective action problems led to the cultural transformation of Sāmoa 800 years ago
Source: PLoS One. 2024 Jun 20;19(6):e0304850. doi: 10.1371/journal.pone.0304850 (PMC11189243; doi:10.1371/journal.pone.0304850)

S1 Figure. Comparison of lidar identified features and density of ground returns. Note that large gaps between features are associated with low ground return density, indicated by light gray to white shading.

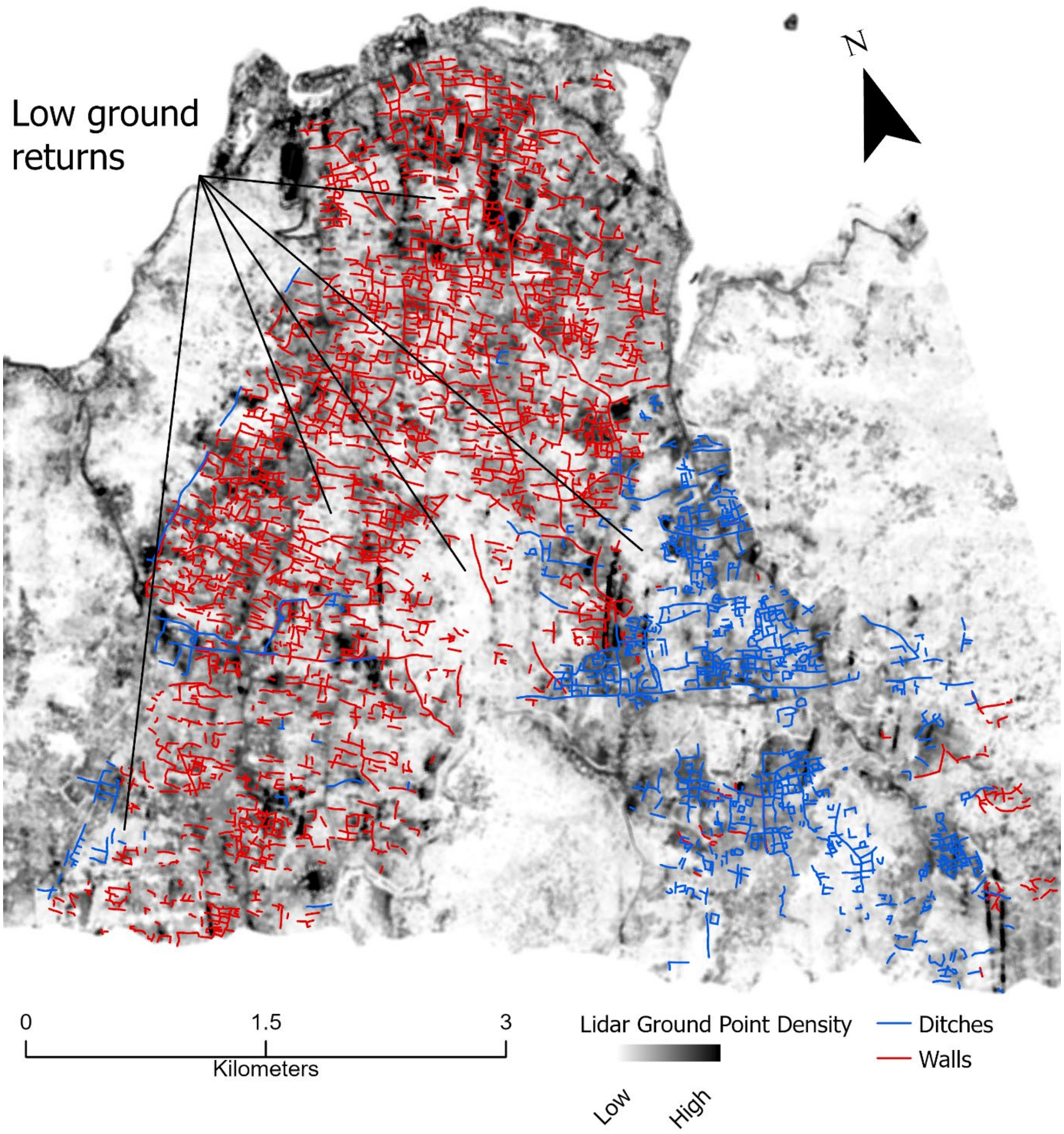

Supplement: S1 Fig — (PDF) [file pone.0304850.s007.pdf]
