# Supplementary material for: Collective action problems led to the cultural transformation of Sāmoa 800 years ago
Source: PLoS One. 2024 Jun 20;19(6):e0304850. doi: 10.1371/journal.pone.0304850 (PMC11189243; doi:10.1371/journal.pone.0304850)

S2 Figure. Base course of rock wall (Feature 4150).

Corners of excavation unit marked by pink flagging tape. Fieldbook in center is approximately 19 cm long. View to west.

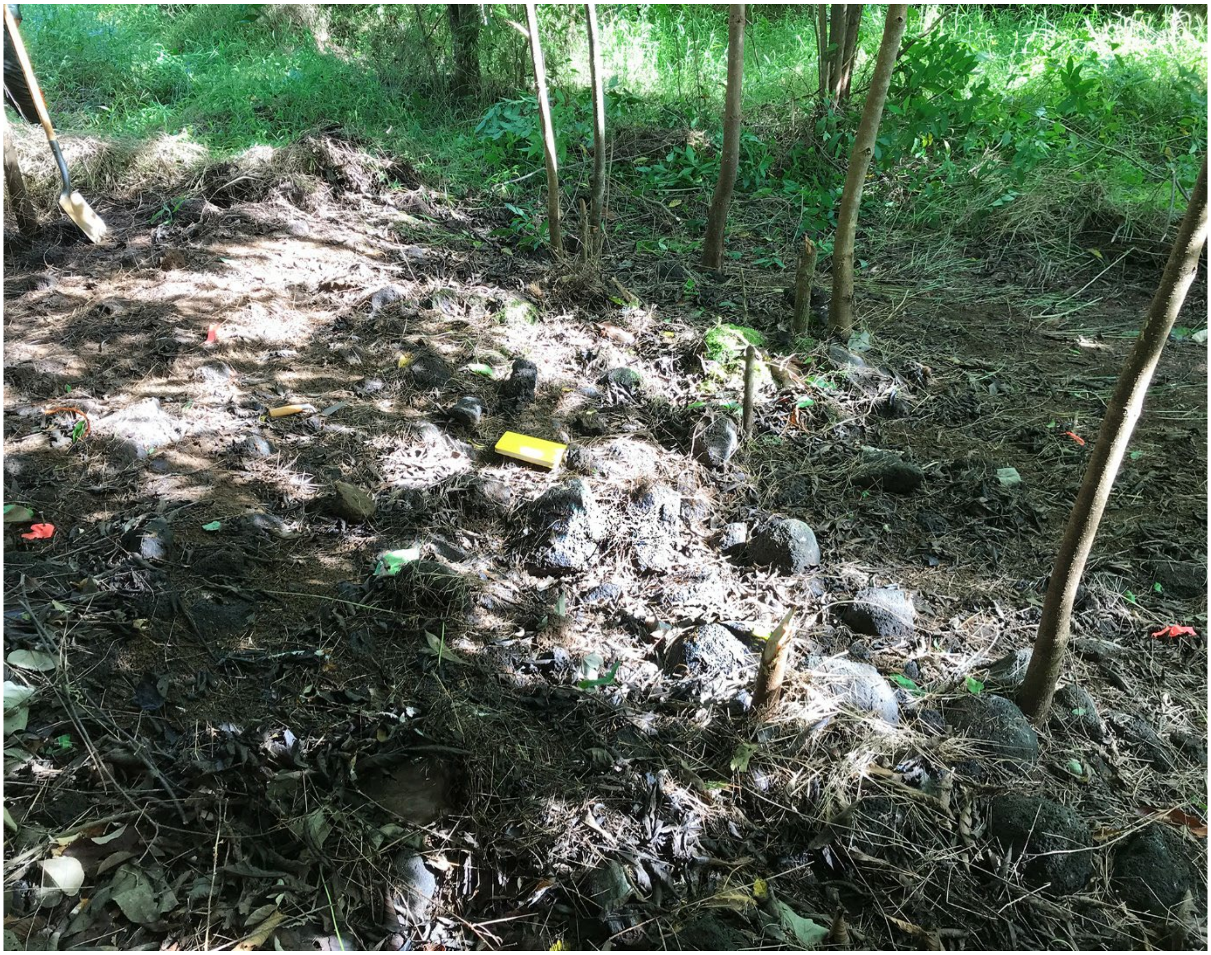

Supplement: S2 Fig — (PDF) [file pone.0304850.s008.pdf]
