# Supplementary material for: Collective action problems led to the cultural transformation of Sāmoa 800 years ago
Source: PLoS One. 2024 Jun 20;19(6):e0304850. doi: 10.1371/journal.pone.0304850 (PMC11189243; doi:10.1371/journal.pone.0304850)
Supplement: S1 Table — (PDF) [file pone.0304850.s009.pdf]

S1 Table. Radiocarbon data for all samples

Village (Site [if available]), Feature	Provenience[1]	Lab No.	Sample Material	$^{13}C/^{12}C$ Ratio ($^{0}/_{00}$)	Conventional Radiocarbon Age (BP)	Error	Calibrated 2 sd age range (BP)*	Deposit interpretation
Falevao D-section cores	FV1, 219-220.5 cmbs	Wk-54994	Plant material		976	17	927-901 (0.33)<br>870-797 (0.62)	Various sediments trapped in Falevao catchment
	FV1, 262.5-264cmbs	Wk-51322	Plant material		4666	24	5465-5319	
	FV19, 190 cmbs	Wk-51320	Plant material		663	24	669-629 (0.49)<br>593-560 (0.47)	
	FV19, 279 cmbs	Wk-54998	Wood/plant material		3504	40	3887-3688 (0.93)<br>3662-3646 (0.03)	
	FV19, 310-311 cmbs	Wk-51321	Plant material		5041	24	5900-5719	
Falevao (Vai inu), stream section 3201	Layer 1, 0-33 cmbs	Wk-48233	*C. nucifera* endocarp		-		109.2 +/- 0.5 % modern	Brown silty clay loam, some fine roots.
	Layer 5, 116-128 cmbs	Wk-48231	*A. moluccana* endocarp		712	22	681-649 (0.91)<br>580-573 (0.44)	Burn layer: dark grey brown sandy clay, some fine roots; includes band of orange oxidised clay peds at top of layer, abundant charcoal
		Wk-48232	*S. ghaeri* seed		561	22	629-592 (0.47)<br>560-527 (0.49)	
	Layer 4, 95-105 cmbs	Wk-48236	*S. ghaeri* seed		672	22	671-635 (0.54)<br>590-562 (0.41)	Burn layer: grey sandy silt, some roots,

Village (Site [if available]), Feature	Provenience[1]	Lab No.	Sample Material	$^{13}C/^{12}C$ Ratio ($^{0}/_{00}$)	Conventional Radiocarbon Age (BP)	Error	Calibrated 2 sd age range (BP)*	Deposit interpretation
Falevao (Fafelo), stream section 3206	Layer 4, 105-115 cmbs	Wk-48234	*A. moluccana* endocarp		713	22	681-650 (0.92) 580-573 (0.039)	frequent river-rolled rock, 3-5 mm diameter, with some >5 mm; water table at 95 cm; abundant charcoal
		Wk-48235	*S. ghaeri* seed		622	22	652-553	
Falefa, large stone wall Feature 1156	Layer 2, 88 cmbd	Beta-549555	*C. nucifera* endocarp	-25.9	-		103.93 +/- 0.39 % modern	Feature layer: brown clay loam containing feature rocks, abrupt lower boundary
	Layer 3, subfeature 1, 83-90 cmbd	Wk-50289	Unidentified charcoal		940	15	912-793	Pre-wall: charcoal-filled basin cut in to layer 3
	Layer 3, 80 cmbd	Wk-50290	Unidentified charcoal		990	15	954-800	Pre-wall: orange-brown, sandy clay loam, weathered basalt rocks, charcoal in top 5 cm
Falefa, large stone wall Feature 1164	Layer 2, 49-58 cmbd	Wk-50286	Unidentified charcoal		604	15	607-548	Feature layer: silty clay containing basal feature rocks, clear lower boundary
	Layer 2, 58-64 cmbd	Wk-50287	Unidentified charcoal		684	21	589-562	
	Layer 3, 64-75 cmbd	Wk-50285	Unidentified charcoal		593	15	641-591	Pre-wall: silty clay, more friable, fewer roots than layer 2
	Layer 3, 64-75 cmbd	Beta-550298	Indeterminate parenchyma	-22.3	610	30	654-585	

Village (Site [if available]), Feature	Provenience[1]	Lab No.	Sample Material	$^{13}C/^{12}C$ Ratio ($^{0}/_{00}$)	Conventional Radiocarbon Age (BP)	Error	Calibrated 2 sd age range (BP)*	Deposit interpretation
	Layer 3, 64-75 cmbd	Beta-550299	*Psychotria* sp. wood charcoal	-29.9	620	30	657-583	
	Layer 3, 73 cmbd	Wk-52090	Unidentified charcoal		844	19	784-690	
	Layer 3, 75-87 cmbd	Beta-550300	*Psychotria* sp. wood charcoal	-28.1	1300	30	1293-1131	
Falefa, single stone wall Feature 3330	Subfeature 1, 65-68 cmbd	Wk-50284	Unidentified charcoal		160	15	283-0	Pre-wall: subfeature is corner of platform within Layer 2
	Subfeature 1, 78 cmbd (adhering to base of subfeature rock)	Wk-50281	Unidentified charcoal		169	15	285-0	
	Subfeature 2, 72-102 cmbd	Beta-549560	Small diameter monocot stem	-27.9	2060	30	2111-1934	Pre-wall: post-hole excavated into layer 2
	Layer 2, 61-65 cmbd	Wk-50283	Unidentified charcoal		1006	18	960-829	Pre-wall: orange-brown sandy clay predating subfeatures 1 and 2
	Layer 2, 65-68 cmbd	Beta-549559	Indeterminate parenchyma	-28.4	1230	30	1269-1066	
	Layer 2, 77-84 cmbd	Beta-549561	Indeterminate parenchyma	-28.5	1240	30	1272-1070	
	Layer 2, 77-84 cmbd	Wk-50282	Unidentified charcoal		812	14	731-685	
Falefa, single stone wall Feature 3335	Layer 2, 40-49 cmbd	Beta-549558	*Calophyllum* sp.	-26.8	430	30	527-335	

Village (Site [if available]), Feature	Provenience[1]	Lab No.	Sample Material	$^{13}C/^{12}C$ Ratio ($^0/_{00}$)	Conventional Radiocarbon Age (BP)	Error	Calibrated 2 sd age range (BP)*	Deposit interpretation
	Layer 2, 49-64 cmbd	Wk-50288	Unidentified charcoal		241	15	308-155	Pre-wall: pale brown silty clay layer with degraded basalt rocks
Falefa, large stone wall Feature 4150	Layer 1, 72 cmbd (adhering to base of feature rock)	Wk-50279	Unidentified charcoal		395	16	503-335	Feature layer: silty clay containing feature rocks, many, very fine roots, clear lower boundary
	Layer 1, 72 cmbd (adhering to base of feature rock)	Wk-52091	Unidentified charcoal		520	19	549-514	
	Layer 2, 63-75 cmbd	Wk-52062	Indeterminate monocot		626	19	653-555	Pre-wall: loose sediment from roots and rock removal; sandy clay loam, very few fine roots
	Layer 2, 75-95 cmbd	Beta-549556	*Xylosma* sp. charcoal	-26.7	1820	30	1860-1630	Pre-wall: sandy clay loam, very few fine roots
	Layer 2, 75-95 cmbd	Beta-549557	*Myristica* sp. Wood charcoal	-26.1	1940	30	1969-1821	
	Layer 2, 75-95 cmbd	Wk-50280	Unidentified wood charcoal		1887	15	1883-1743	
Saoluafata, single stone wall Feature 2084	Layer 2, 58 cmbd	Wk-52052	*Pipturus* sp.		390	19	504-330	Pre-wall: charcoal adhering to base of rock in sandy clay loam, very few find and coarse roots
	Layer 2, 58 cmbd	Wk-52053	*Morinda citrifolia*		992	18	956-799	

Village (Site [if available]), Feature	Provenience[1]	Lab No.	Sample Material	$^{13}C/^{12}C$ Ratio ($^{0}/_{00}$)	Conventional Radiocarbon Age (BP)	Error	Calibrated 2 sd age range (BP)*	Deposit interpretation
Saoluafata, single stone wall Feature 2086	Layer 2, 40 cmbd	Wk-52055	*Thespesia populnea*		649	18	660-559	Feature layer: sandy clay, very few very fine to medium roots, gradual smooth boundary
	Layer 2, 44 cmbd	Wk-52054	Indeterminate parenchyma		394	18	504-333	Feature layer: charcoal adhering to bottom of feature base rock, gradual smooth boundary
	Layer 3, 62-70 cmbd	Wk-52056	*Fagraea berteroana*		987	18	953-798	Pre-wall: sandy clay loam, very few fine roots
	Layer 3, 62-70 cmbd	Wk-52057	*Tarenna sambucina*		2035	19	2045-1896	
Saoluafata, large stone wall Feature 4108	Layer 2, 42-56 cmbd	Wk-52058	*Pipturus* sp.		582	19	638-541	Feature layer: very few fine to medium roots, sandy clay
	Layer 2, 56-63 cmbd	Wk-52059	Indeterminate angiosperm twig		568	19	630-531	
	Layer 2, 56-63 cmbd	Wk-52060	*Pipturus* sp.		568	19	630-531	
	Layer 2, 56-63 cmbd	Wk-52061	Indeterminate monocot, small dia. stem		567	18	629-533	
	Layer 1, 11-33 cmbd	Wk-54997	*Glochidion* sp.		564	18	628-594 (0.5)<br>559-532 (0.46)	Feature layer: common fine roots, sandy clay

Village (Site [if available]), Feature	Provenience[1]	Lab No.	Sample Material	$^{13}C/^{12}C$ Ratio ($^{0}/_{00}$)	Conventional Radiocarbon Age (BP)	Error	Calibrated 2 sd age range (BP)*	Deposit interpretation
Saoluafata, single stone wall Feature 5021	Layer 2, 50 cmbd	Wk-52063	*Omalanthus nutans*		992	18	956-799	Pre-wall: very few very fine & medium roots, sandy clay
	Layer 3, 58 cmbd	Wk-52064	Indeterminate angiosperm		1051	18	973-923	Pre-wall: sandy clay
Saoluafata, large stone wall Feature 5023	Layer 1, 32-43 cmbd	Wk-52065	Indeterminate monocot		171	18	288-0	Feature layer: few very fine to fine roots, very few medium roots, sandy clay
	Layer 1, 32-43 cmbd	Wk-52066	Indeterminate monocot		542	17	623-523	
	Layer 1, 43-56 cmbd	Wk-52067	Indeterminate monocot stem		372	18	495-325	
	Layer 1, 43-56 cmbd	Wk-52068	Indeterminate bark		350	18	479-316	
	Layer 1, 43-56 cmbd	Wk-52069	Indeterminate monocot stem		346	19	476-316	
Saoluafata, large stone wall Feature 5052	Layer 1, 43-55 cmbd	Wk-52070	Indeterminate monocot stem		228	18	309-150	Feature layer: few, very fine roots & very few fine roots & coarse roots, silty clay
	Layer 1, 43-55 cmbd	Wk-52071	*Cocos nucifera* endocarp		324	18	455-311	
	Layer 1, 43-55 cmbd	Wk-52072	*Cocos nucifera* endocarp		326	18	456-312	
	Layer 2, 55-61 cmbd	Wk-52073	Indeterminate, possible seed casing		189	19	290-0	Pre-wall: very few coarse roots, clay loam

Village (Site [if available]), Feature	Provenience[1]	Lab No.	Sample Material	$^{13}C/^{12}C$ Ratio ($^{0}/_{00}$)	Conventional Radiocarbon Age (BP)	Error	Calibrated 2 sd age range (BP)*	Deposit interpretation
	Layer 2, 55-61 cmbd	Wk-52074	Indeterminate monocot stem		339	18	470-315	
Saoluafata, single stone wall Feature 5058	Layer 1, 57-75 cmbd	Wk-52079	*Omalanthus nutans*, small dia. branch		403	19	507-335	Feature layer: many very fine to fine roots, common coarse roots, silty clay
	Layer 1, 57-75 cmbd	Wk-52080	*Kleinhovia hospita*, small dia. branch		389	19	503-330	
	Layer 2, 88-93 cmbd	Wk-54996	Unidentified wood charcoal		467	18	528-499	Pre-wall: common coarse roots, silty clay
Saoluafata, single stone wall Feature 5063	Layer 1 & 2, 42-63 cmbd	Wk-52082	*Mirystica* sp.		590	18	640-545	Pre-wall: samples 20 cm below feature lower boundary in silty clay, common coarse & few fine roots (Lay. 1) and sandy clay, very few very fine roots (Lay. 2)
	Layer 1 & 2, 42-63 cmbd	Wk-52083	*Pipiturus* sp.		353	17	479-317	
	Layer 1 & 2, 42-63 cmbd	Wk-52084	*O. nutans*		592	18	643-545	
Saoluafata,, single stone wall Feature 6000	Layer 1, 35-50 cmbd	Wk-52085	Indeterminate monocot		442	19	522-484	Feature layer: few very fine roots, very few fine to medium roots, clay loam
	Layer 1, 35-50 cmbd	Wk-52086	Indeterminate angiosperm, small dia.		411	17	510-344	
	Layer 1, 50-59 cmbd	Wk-52087	Indeterminate angiosperm, small dia.		441	19	521-483	

Village (Site [if available]), Feature	Provenience[1]	Lab No.	Sample Material	$^{13}C/^{12}C$ Ratio ($^0/_{00}$)	Conventional Radiocarbon Age (BP)	Error	Calibrated 2 sd age range (BP)*	Deposit interpretation
	Layer 1, 50-59 cmbd	Wk-52088	Indeterminate angiosperm, small dia.		453	19	524-493	
	Layer 2, 59-70	Wk-52089	Indeterminate angiosperm, small dia.		444	19	523-485	Pre-wall: very few fine roots, sandy clay

[1] Single depth (i.e., not range) indicates point provenience sample

* August 2021 using Oxcal 4.4
