# Supplementary material for: Collective action problems led to the cultural transformation of Sāmoa 800 years ago
Source: PLoS One. 2024 Jun 20;19(6):e0304850. doi: 10.1371/journal.pone.0304850 (PMC11189243; doi:10.1371/journal.pone.0304850)
Supplement: S1 Code — (DOCX) [file pone.0304850.s010.docx]

**S1 Code. Oxcal code for Bayesian chronologiacl models incorporating stratigraphically determined relative constuction order.** Author: Seth Qutinus.

Plot()

{

MCMC_Sample("FalefaWalls",25,200000)

{

Outlier_Model("Charcoal", Exp(1,-10,0), U(0,2.3), t);

Phase("Land Use Activities")

{

Sequence("Burn Layers")

{

Boundary("Beginning of Burn");

Phase("Features 3201-3206")

{

R_Date("Wk-48231", 712, 22);

R_Date("Wk-48234", 713, 22);

};

Boundary("End of Burn Layer");

Before(1950)

{

C_Date(1950, 1);

};

};

Sequence("Double Wall 1156")

{

Boundary("Beginning of 1156");

Phase("Double Wall 1156 TPQ")

{

R_Date("Wk-50289", 940, 15)

{

Outlier("Charcoal", 1);

};

R_Date("Wk-50290", 990, 15)

{

Outlier("Charcoal", 1);

};

};

Boundary("Double Wall 1156 Construction");

Before(1950)

{

C_Date(1950, 1);

};

};

Sequence("Double Wall 1164")

{

Boundary("Beginning of 1164");

Phase("Double Wall 1164 Layer III")

{

R_Date("Wk-52090", 844, 19)

{

Outlier("Charcoal", 1);

};

R_Date("Wk-50285", 593, 15)

{

Outlier("Charcoal", 1);

};

R_Date("Beta-550300", 1300, 30);

R_Date("Beta-550298", 610, 30)

{

Outlier("Charcoal", 1);

};

R_Date("Beta-550299", 620, 30);

};

Phase("Double Wall 1164 Layer II")

{

R_Date("Wk-50287", 684, 21)

{

Outlier("Charcoal", 1);

};

R_Date("Wk-50286", 604, 15)

{

Outlier("Charcoal", 1);

};

};

Boundary("Double Wall 1164 Construction");

Before(1950)

{

C_Date(1950, 1);

};

};

Sequence("Double Wall 4150")

{

Boundary("Beginning of 4150");

Phase("Double Wall 4150 Layer II")

{

R_Date("Wk-50280", 1887, 15)

{

Outlier("Charcoal", 1);

};

R_Date("Beta-549556", 1820, 30);

R_Date("Beta-549557", 1940, 30)

{

Outlier("Charcoal", 1);

};

R_Date("Wk-52062", 626, 19)

{

Outlier("Charcoal", 1);

};

};

Phase("Double Wall 4150 Layer I")

{

R_Date("Wk-50279", 395, 16)

{

Outlier("Charcoal", 1);

};

R_Date("Wk-50291", 520, 19)

{

Outlier("Charcoal", 1);

};

};

Boundary("Double Wall 4150 Construction");

Phase("Abutting Relationships")

{

Date("=Single Wall 3330 Construction");

Date("=Single Wall 3335 Construction");

};

Before(1950)

{

C_Date(1950, 1);

};

};

Sequence("Single Wall 3330")

{

Boundary("Beginning of 3330");

Phase("Single Wall 3330 Layer II")

{

R_Date("Wk-50282", 812, 14)

{

Outlier("Charcoal", 1);

};

R_Date("Wk-50283", 1006, 18)

{

Outlier("Charcoal", 1);

};

R_Date("Beta-549559", 1230, 30)

{

Outlier("Charcoal", 1);

};

R_Date("Beta-549561", 1240, 30)

{

Outlier("Charcoal", 1);

};

};

Phase("Single Wall 3330 Feature I")

{

R_Date("Wk-50281", 169, 15)

{

Outlier("Charcoal", 1);

};

R_Date("Wk-50284", 160, 15)

{

Outlier("Charcoal", 1);

};

};

Boundary("Single Wall 3330 Construction");

Before(1950)

{

C_Date(1950, 1);

};

};

Sequence("Single Wall 3335")

{

Boundary("Beginning of 3335");

Phase("Single Wall 3335 TPQ")

{

R_Date("Wk-50288", 241, 15)

{

Outlier("Charcoal", 1);

};

R_Date("Beta-549558", 430, 30)

{

Outlier("Charcoal", 1);

};

};

Boundary("Single Wall 3335 Construction");

Before(1950)

{

C_Date(1950, 1);

};

};

Sequence("Single Wall 2084")

{

Boundary("Beginning of 2084");

Phase("Single Wall 2084 TPQ")

{

R_Date("Wk-52052", 390, 19);

R_Date("Wk-52053", 992, 18)

{

Outlier("Charcoal", 1);

};

};

Boundary("Single Wall 2084 Construction");

Before(1950)

{

C_Date(1950, 1);

};

};

Sequence("Single Wall 2086")

{

Boundary("Beginning of 2086");

Phase("Single Wall 2086 Layer III")

{

R_Date("Wk-52057", 2035, 19)

{

Outlier("Charcoal", 1);

};

R_Date("Wk-52056", 987, 18)

{

Outlier("Charcoal", 1);

};

};

Phase("Single Wall 2086 Layer II")

{

R_Date("Wk-52054", 394, 18)

{

Outlier("Charcoal", 1);

};

R_Date("Wk-52055", 649, 18)

{

Outlier("Charcoal", 1);

};

};

Boundary("Single Wall 2086 Construction");

Before(1950)

{

C_Date(1950, 1);

};

};

Sequence("Double Wall 4108")

{

Boundary("Beginning of 4108");

Phase("Double Wall 4108 TPQ")

{

R_Date("Wk-52058", 582, 19);

R_Date("Wk-52059", 568, 19);

R_Date("Wk-52060", 568, 19);

R_Date("Wk-52061", 567, 18);

};

Boundary("Double Wall 4108 Construction");

Date("=Double Wall 5023 Construction");

Before(1950)

{

C_Date(1950, 1);

};

};

Sequence("Single Wall 5021")

{

Boundary("Beginning of 5021");

Phase("Single Wall 5021 Layer III")

{

R_Date("Wk-52064", 1051, 18)

{

Outlier("Charcoal", 1);

};

};

Phase("Single Wall 5021 Layer II")

{

R_Date("Wk-52063", 992, 18);

};

Phase("Single Wall 5021 Layer I")

{

R_Date("Wk-54997",564,18);

};

Boundary("Single Wall 5021 Construction");

Before(1950)

{

C_Date(1950, 1);

};

};

Sequence("Double Wall 5023")

{

Boundary("Beginning of 5023");

Phase("Double Wall 5052 TPQ")

{

R_Date("Wk-52065", 171, 18)

{

Outlier("Charcoal", 1);

};

R_Date("Wk-52066", 542, 17)

{

Outlier("Charcoal", 1);

};

R_Date("Wk-52067", 372, 18)

{

Outlier("Charcoal", 1);

};

R_Date("Wk-52068", 350, 18);

R_Date("Wk-52069", 346, 19)

{

Outlier("Charcoal", 1);

};

};

Boundary("Double Wall 5023 Construction");

Date("=Single Wall 5021 Construction");

Before(1950)

{

C_Date(1950, 1);

};

};

Sequence("Double Wall 5052")

{

Boundary("Beginning of 5052");

Phase("Double Wall 5052 Layer II")

{

R_Date("Wk-52074", 339, 18)

{

Outlier("Charcoal", 1);

};

};

Phase("Double Wall 5052 Layer I")

{

R_Date("Wk-52072", 326, 18);

R_Date("Wk-52071", 324, 18);

R_Date("Wk-52070", 228, 18)

{

Outlier("Charcoal", 1);

};

R_Date("Wk-52073", 189, 19);

};

Boundary("Double Wall 5052 Construction");

Date("=Single Wall 5058 Construction");

Before(1950)

{

C_Date(1950, 1);

};

};

Sequence("Single Wall 5058")

{

Boundary("Beginning of 5058");

Phase("Wall 5058 Layer II")

{

R_Date("Wk-54996",467,18);

};

Phase("Wall 5058 TPQ")

{

R_Date("Wk-52080", 389, 19);

R_Date("Wk-52079", 403, 19);

};

Boundary("Single Wall 5058 Construction");

Before(1950)

{

C_Date(1950, 1);

};

};

Sequence("Single Wall 5063")

{

Boundary("Beginning of 5063");

Phase("Wall 5063 TPQ")

{

R_Date("Wk-52082", 590, 18)

{

Outlier("Charcoal", 1);

};

R_Date("Wk-52083", 353, 17);

R_Date("Wk-52084", 592, 18)

{

Outlier("Charcoal", 1);

};

};

Boundary("Single Wall 5063 Construction");

Before(1950)

{

C_Date(1950, 1);

};

};

Sequence("Single Wall 6000")

{

Boundary("Beginning of 6000");

Phase("Single Wall 6000 Layer II")

{

R_Date("Wk-52089", 444, 19);

};

Phase("Single Wall 6000 Layer I")

{

R_Date("Wk-52088", 453, 19);

R_Date("Wk-52087", 441, 19);

R_Date("Wk-52086", 411, 17);

R_Date("Wk-52085", 442, 19)

{

Outlier("Charcoal", 1);

};

};

Boundary("Single Wall 6000 Construction");

Before(1950)

{

C_Date(1950, 1);

};

};

};

};

};
